# Mental-Imagery-Based Mnemonic Training: A New Kind of Cognitive Training

**DOI:** 10.3389/fpsyg.2021.740829

**Published:** 2022-02-09

**Authors:** Xiaoyu Luan, Yayoi Kawasaki, Qi Chen, Eriko Sugimori

**Affiliations:** ^1^Graduate School of Human Sciences, Waseda University, Tokorozawa, Japan; ^2^Faculty of Human Sciences, Waseda University, Tokorozawa, Japan; ^3^Medical College, Yangzhou University, Yangzhou, China

**Keywords:** cognitive training, fluid intelligence, long-term memory, mental imagery, mnemonic, children

## Abstract

We investigated the immediate and maintenance effects of mental-imagery-based mnemonic training on improving youths’ working memory, long-term memory, arithmetic and spatial abilities, and fluid intelligence. In Experiment 1, 26 Chinese participants (15 boys, 11 girls) aged 10–16 years were divided into an experimental group that received 8 days of mental-imagery-based mnemonic training and a no-contact control group. Participants completed pre-, post-, and three follow-up tests (3, 6, and 12 months after the pre-test). In Experiment 2, 54 Chinese children (28 boys, 26 girls), all 12 years old, were divided into experimental and control groups. Participants completed pre-, post-, and follow-up tests (three months after the pre-test). Results showed that the training significantly affected long-term memory-related task performance but no effects were observed on working memory, arithmetic or spatial ability, or fluid intelligence-related tasks. Moreover, the effect of the training on long-term memory lasted up to one year; the more frequently the training was used, the more effective it was. A content analysis of the feedback submitted by parents of participants in Experiment 2 three months after the training showed that the children used the strategy more for memorizing content such as Chinese and English, as well as for musical scores. Furthermore, there was also the possibility that the training improved abilities and academic performance such as concentration and math performance. Our results provide a basis for the further exploration of mental-imagery-based mnemonic training as a novel training modality.

## Introduction

Cognitive training encompasses a series of training programs aimed at improving or maintaining one’s cognitive abilities (Simons et al., 2016), particularly general fluid intelligence (Gf; Tsubomi et al., 2019). In contrast to crystallized intelligence, which includes knowledge and experience, Gf is a collection of abilities that allows people to solve novel cognitive problems and adapt to new situations without relying on previous knowledge (Carpenter et al., 1990). Gf is highly predictive of academic and professional outcomes, including attention control, memory, problem solving, and reading (Jaeggi et al., 2008). Cognitive training research has been conducted for centuries (Katz et al., 2018), including on working memory (WM) training (Au et al., 2015), neuro-feedback training (Gruzelier, 2014), exercise training (Kramer and Colcombe, 2018), and music training (Schellenberg, 2011).

Existing cognitive training research tends to focus on two aspects of the commonality between trained capacities and Gf. For example, in WM training, research has explored whether WM capacity can be enhanced by WM training (McNamara and Scott, 2001; Klingberg, 2010) and whether WM training can improve cognitive abilities such as near and far transfer of Gf (Sala and Gobet, 2017b; Gathercole et al., 2019). Learning transfer occurs when skills acquired through particular behaviors of cognitive training are generalized to other fields (Sala and Gobet, 2017a). Near transfer refers to the transfer of skills among closely related tasks; for example, training on the n-back task leads to higher scores on WM tasks than on training tasks such as the digit span task. Far transfer refers to the transfer that occurs among tasks with a weak connection, such as higher-order cognitive tasks (e.g., mental rotation tasks) other than WM tasks (Sala and Gobet, 2017a). Numerous studies have demonstrated the attentional benefits of cognitive training (Chein and Morrison, 2010; Jaeggi et al., 2011; Karbach and Verhaeghen, 2014), although many have failed to find significant benefits (Harrison et al., 2013; Melby-Lervåg et al., 2016; Simons et al., 2016). Nevertheless, a growing body of literature exploring how cognitive training transfers to improve Gf or individuals’ daily lives shows that the possibility of cognitive training is receiving increasing attention.

Referring to Table 1, there are three limitations of current cognitive training in general. First, although studies have shown that cognitive training can produce some effects in terms of near transfer, such effects are strongly stimulus-type restricted, and thus are somewhat far from being applied to real-life situations (e.g., Lustig et al., 2009). Second, with regard to far transfer, as shown above, while some studies found that several cognitive trainings can produce far transfer, there is also a considerable amount of counter evidence; hence, their real effects are still under debate. Third, many training programs require the use of computers, equipment such as brainwave meters, musical instruments, or sports venues; these external necessities limit the possibility of long-term self-training in the daily life of the user. Furthermore, while some studies point to the need for long-term research (e.g., Morrison and Chein, 2011), most studies still focus only on the immediate effects of training after completion (e.g., Kuhn and Holling, 2014; Studer-Luethi et al., 2016), which presents a challenge to our understanding of the true role of these trainings in our lives.

**TABLE 1:** Benefits and limitations of existing cognitive trainings.

Training types	Training contents	Benefits	Limitations
**WM training**			
(a) Strategy training	Enhance WM capacity through effective encoding, maintenance, or retrieval techniques (e.g., the Method of Loci)	(1) Increase performance on LTM tasks	(1) Seldom used to contribute to cognitive enhancement (2) Low effectiveness in real-life applications (e.g., academic performance)
(b) Core training	Enhance the WM function or capacity itself (e.g., N-back)	(1) Increasing performance on WM tasks (2) May enhance other cognitive abilities (e.g., Gf)	(1) Need to use additional machines (e.g., computers) (2) Still no consensus on the real role that core training as cognitive training can play in real-life applications
Neuro-feedback training	An operant conditioning paradigm in which the user learns to influence the electrical activity of the brain by providing sensory feedback	(1) May enhance other cognitive abilities (e.g., Gf)	(1) Need to use additional machines (e.g., computers) (2) Need to record the user’s brain waves (3) Lack of long-term research (4) Still no consensus on the real role that core training as cognitive training can play in real-life applications
Exercise training	Exercise improves cognitive abilities through cellular and neurochemical changes	(1) May enhance other cognitive abilities (e.g., Gf)	(1) Consistent training required (2) Need for a proper place to exercise (3) Not all exercise is effective
Music training	Improve cognitive abilities such as operating memory through music training	(1) May enhance cognitive abilities related to auditory information processing	(1) Consistent training required (2) May be affected by external factors such as instruments (3) Most studies lack the reliability to test causal hypotheses

*WM, working memory; LTM, long-term memory; Gf, fluid intelligence.*

Considering the benefits and limitations of the various types of existing cognitive training, the purpose of this study is to propose a cognitive training that has no limitation of stimulus types in near transfer and can facilitate far transfer, improves users’ cognitive abilities more comprehensively, and does not require additional equipment or space, allowing users to easily and consistently apply it in their daily lives—mental-imagery-based mnemonic training (MIBMT), and validate its effectiveness. We position MIBMT as a method that can be “used” rather than “exercised,” that is, we hope that once users have mastered MIBMT, they do not need to dedicate a certain amount of time to daily training (e.g., core training, exercise training) to maintain its effects; rather, they can apply MIBMT directly to their daily studies or work, such as second-language learning.

## Mental-Imagery-Based Mnemonic Training: Contents and Theoretical Basis

Mental-imagery-based mnemonic training is a generalized program designed to enhance WM and long-term memory (LTM) performance and improve yield transfer and Gf, using verbal and non-verbal mental-imagery mnemonic strategies in collaboration with mindfulness meditative practices. MIBMT consists of three elements: a short practice of mindfulness at the beginning of the training to improve participants’ attention, conversion of the target stimulus into mental imagery (MI) after the stimulus is presented auditorily/visually—the participant maintains it in their mind for the necessary encoding operations, and recollection of the target stimulus in reverse order.

### Theoretical Basis

#### Mindfulness Practice

Mindfulness practice focuses on the present moment in a non-reactive way, and includes the seven attitudinal factors of “non-judging, patience, a beginner’s mind, trust, non-striving, acceptance, and letting go” (Kabat-Zinn and Hanh, 2009, p. 32). Studies have shown that experienced mindfulness users have better executive attentional performance (van den Hurk et al., 2010) and reduced mind wandering compared to those without mindfulness experience (Mrazek et al., 2012, 2013). Further, differences in attention can explain individual differences in WM capacity to some extent (Wiemers et al., 2019). Thus, mindfulness can extend WM capacity by limiting irrelevant information or expanding the amount of information that can be accommodated in WM (e.g., van Vugt and Jha, 2011).

Many studies have demonstrated the effectiveness of mindfulness training in adolescents (Quach et al., 2016), highlighting its effects during short-term training (Zeidan et al., 2010). Therefore, given the theoretical links among mindfulness practice, WM, attention, and mind wandering, using a short period of mindfulness practice initially in MIBMT may help participants focus their attention and reduce mind wandering, further improving training effectiveness.

#### Mental Imagery

Marschark et al. (1987) clearly showed that MI has a functional role in human cognition and that it contributes directly or indirectly to memory, list learning, language comprehension, and mind manipulation. Specifically, MI overlaps with many of the same mechanisms used in visual perception and with the central executive system in WM processing, such as information manipulation, and it plays an irreplaceable role in LTM. For example, concrete words are easier to recall than abstract words because MI can help form more characteristically cohesive encodings, and thus it provides additional retrieval avenues (Marschark et al., 1987). At the neural level, MI and WM share a common characterization function in the early visual cortex (Albers et al., 2013), and two-thirds of the brain’s regions that are activated during visual imagery processing coincide with the areas activated during visual perception (Kosslyn et al., 1997).

It is theoretically possible to improve the performance of WM and LTM by exploiting MI and intermediating the relationship between WM and LTM with Gf to further trigger a far transfer. Kosslyn (2005) argued that representations of LTM are inherently multimodal, linking visual, spatial, auditory, and tactile types of information together to build the structure of objects in memory. This coincides with a requirement of MIBMT—that participants determine multi-sensory characteristics of the target stimuli, such as how they look or feel, and create MI to enhance memorization.

In addition, according to the oral presentation effect, better results can be obtained when stimuli are presented for aural perception while using MI-based mnemonics, such as the method of loci (Cornoldi and de Beni, 1991; Moè and de Beni, 2005) and the cue-word method (de Beni and Moè, 2003). With this feature in mind, MIBMT presents stimuli aurally at the beginning of training. When users can use this strategy more consistently, the alternating presentation of audio and visual text helps them apply it to different real-life situations once the training sessions have concluded.

#### Recollection of the Target Stimulus in Reverse Order

One of the most important attributes of MIBMT is the third element that requires the user to maintain target stimuli in the form of MI and subsequently recall it in reverse order to enhance the processing level of encoding. Marschark et al. (1987) mentioned that abnormal sentences or jumbled prose, for example, allow attention to be focused on each individual word, while meaningful sentences are usually treated as a whole and result in errors generated by the top-down system during subsequent recalls (e.g., recalling “learning English is hard” as “it is hard to learn English”).

Furthermore, the textual anomalies produced by the act of recalling in reverse order improve participants’ grasp of the information related to each individual word/character, such as visual or spatial characteristics, and leads to a conscious calling into mind, or gaze, of the MI the participant holds in their mind. Reverse recall is likely to improve the accuracy of a person’s gaze, while a higher fidelity gaze can also improve recall accuracy (Laeng et al., 2014). Further, when people retrieve information from memory, they tend to anchor the information in the location where it appears during encoding (Scholz et al., 2015). Thus, an MI constructed with a high level of encoding and accuracy may help reproduce the exact position of the elements of the encoded stimulus during the retrieval period, thereby facilitating memory performance.

## Research Objectives

Sala and Gobet (2017a) argued that since the probability of far-transfer occurrence is negatively related to the level of expertise in the discipline, theoretically, children are the ideal population to test the occurrence of far transfer. Therefore, we chose typically developing children as participants to explore the effects of MIBMT.

As mentioned above, the purpose of this study is to propose MIBMT, a cognitive training that aims to improve users’ cognitive abilities and can be easily and consistently used in daily life, and to experimentally test its practical effects. Therefore, considering the research on cognitive training to date, we examined the effects of MIBMT in the following three main aspects. First, we examined the scope of the training transfer. Specifically, digit span and non-word recall tasks were used to measure near transfer, and arithmetic and mental rotation tasks were used to measure far transfer. According to Shipstead et al. (2012), in many studies, near transfer to WM has been measured by simple digit span tasks, as improvement in WM capacity is often associated with short-term memory-related tasks. We also used a simple digit span task as a measure of WM storage capacity. Considering that one of the important characteristics of MIBMT is to focus on the extrinsic features of the stimulus instead of semantic processing, we chose a non-word as the retrieval object when selecting the LTM-related task (non-word recall task) to maximize the exploration of the impact of MIBMT on LTM. Regarding far transfer, WM capacity is associated with adolescents’ mathematical ability (Gathercole et al., 2004), and WM training is likely to improve children’s basic arithmetic skills (Sala and Gobet, 2017a). Thus, the arithmetic skills task has been used as one of the measures of the effects of far transfer in many studies (van der Molen et al., 2010). We also used the mental rotation task—a typical method to test spatial capabilities (Moreau, 2013).

Second, we assessed the maintenance of training benefits, hypothesizing that the benefits produced by MIBMT would be somewhat maintainable. Most studies have focused more on the immediate effects of cognitive training, and research on the effects of (even intermittent) maintenance training (Morrison and Chein, 2011) is scarce. Therefore, it is important to study the immediate and ongoing benefits of MIBMT.

Third, we explored the effect of individual differences on MIBMT effects, hypothesizing that the frequency of use of MIBMT by the experimental group after training would impact the effects of MIBMT. Since MIBMT is positioned as a mnemonic that can be applied to all aspects of life learning, the experimental group was encouraged to use this strategy actively in their daily lives after the training; the frequency of spontaneous use of MIBMT in users’ daily lives represents, to some extent, the level of recognition of MIBMT, the motivation to use it, and the level of proficiency in using it. Therefore, we assumed that the frequency of post-training MIBMT usage would also impact the training effect, and individuals who actively use it may have better maintenance results than their counterparts.

We conducted two experiments (Table 2). In Experiment 1, we evaluated the transfer effect, sustainability, and potential factors affecting the effectiveness of MIBMT. In Experiment 2, based on the results obtained in Experiment 1, the test contents were modified in four ways to further examine the effects and influencing factors of MIBMT. First, the digit span task was changed to a Stroop task. Second, a Raven’s Standard Progressive Matrices (SPM) task was added to measure users’ Gf. Third, we limited the experimental participants to sixth graders and changed the duration of the experiment from one year to three months. Finally, we conducted a contents analysis of the feedback submitted by parents of participants in Experiment 2 to investigate the impact of MIBMT on daily life.

**TABLE 2:** Overview of the experiments.

Research Objectives		Tasks	Experiment 1	Experiment 2
Scope of Training Transfer	Near Transfer	WM	Digit Span Task	Stroop Task
		LTM	Non-words Recalling Task	
	Far Transfer	Mathematical Ability	Arithmetic Skills Task	
		Spatial Ability	Mental Rotation Task	
		Gf	None	Raven’s Standard Progressive Matrices (SPM) task
Maintenance of Training Benefits		Duration of the Experiment	1 Year (5 Tests)	3 Months (3 Tests)
Potential Factors Affecting the Effect of MIBMT		The Frequency of Using MIBMT	Five-point Likert Scale	
Impact of MIBMT on Daily Life		Feedback from Parents of Experimental Group Participants	None	Contents Analysis

*WM, working memory; LTM, long-term memory; Gf, fluid Intelligence; MIBMT, mental-imagery-based mnemonic training.*

## Experiment 1

### Ethical Approval

This study was approved by the Ethics Review Committee on Research with Human Participants of Waseda University (no. 2019-152).

### Participants

From September 2019 to September 2020, 26 elementary- and middle-school children between 10 and 16 years old from Yangzhou, Jiangsu Province, China participated in this study. Eighteen participants (11 boys, 7 girls) were in the experimental group (mean age = 11.78 years; *SD* = 1.83), and eight participants (four boys, four girls) were in the control group (mean age = 11.63 years; *SD* = 1.92). To recruit participants, we advertised in SNS groups (WeChat) for parents of elementary and middle-school students. After asking participants if they had eight consecutive free days, we assigned those who had time to the experimental group and the rest to the control group. Participants in the control group were not informed of anything related to MIBMT. All participants were native Chinese speakers and had no previous events or illnesses that we believed would affect training effectiveness or test performance. All parents provided written consent for their children to participate in the study, and an oral assent was obtained from the children. No other exclusion criteria were used in the recruitment. Participants were free to choose whether to participate; moreover, they were informed that they could withdraw from the experiment at any time and that their data would be destroyed. Each participant was provided with an evaluation report after the experiment. Participation was voluntary. To ensure confidentiality and anonymity of the data, we used only the participant number during the experiment.

### Materials

All tasks were originally designed by the primary investigator and ran on E-prime 2.0 (Psychology Software Tools; Washington, United States). The tasks of each test (pre-, post-, and follow-up) used different stimulus questions derived under the same rule/source, which means that all participants completed the same tasks in the five tests; however, the questions differed between tasks.

### Near-Transfer Tasks

#### Digit Span Task

In this test, the screen presented successive integers from 0 to 9 in random order at a speed of one digit per 700 ms, with a dialog box appearing when the last digit of each span disappeared from the screen. The examinee used the keyboard to enter the recalled digit list into the dialog box before submitting it; no time limit was set for entry. The digit span started at five digits and increased gradually, and each span comprised two turns; when at least one turn represented a successful recall, the span would increase by one digit. No upper limit was set for the span, and the highest number attained by participants was 15 digits. The test ended when the participant could not recall the order for either turn in the same span. In each span, one point was counted if both inputs were correct, 0.5 points if only one was correct, and 0 points if both were incorrect. Four points were given at the beginning of this task as a basic score, which meant that even if a participant failed all the questions in this task, they would have four points. If a participant reached a larger span, one point was added for every passed sequence, even if only one of the spans was correct. For example, when a participant failed both times in an eight-digit span round and succeeded only once in the preceding seven-digit span round, their digit span score was 6.5, regardless of whether they succeeded once or twice in the six-digit span round or in preceding rounds (Table 3).

**TABLE 3:** Example of scoring in the digit span task.

Participant number		No. 1		No. 2		No. 3	

Basis score		4		4		4	
Level	Turn	Answer	Point	Answer	Point	Answer	Point
5	1	R	1	R	1	R	1
	2	R		R		W	
6	1	R	1	R	1	W	1
	2	R		R		R	
7	1	R	0.5	W	0	R	0.5
	2	R	0.5	R	0.5	W	0
8	1	W	0	W	0	W	0
	2	W	0	W	0	W	0
**Final score**		**7**		**6.5**		**6.5**	

*R, right answer; W, wrong answer.*

#### Non-word Recall Task

The non-word recall task was used to measure the participants’ LTM ability. Based on Ding’s (2009) method, we constructed 16 non-words: eight three-character non-words (e.g., 更元就) and eight four-character non-words (e.g., 退吴看跳). None of the characters exceeded the range of characters known to participants. Non-words were presented visually, with each word appearing on the screen for 5000 ms in sequence with a blank screen for 3000 ms between words. After all stimuli were shown, participants were asked to move on to other tasks (e.g., digit span), ensuring a 15-min interval between retrieval and stimulus presentation. Fifteen minutes after the end of the stimulus presentation, participants were asked to recall the stimuli in any order and write them down on paper.

Non-word recall task scores were calculated for the successful recall of each character rather than each whole non-word. Participants were not informed of the scoring method, as the memorization of overall textual content is a common occurrence in everyday life, and we did not want participants to attempt consciously to achieve high scores on their recall of individual characters but rather to focus on memorizing all non-words. In this way, we attempted to simulate the MIBMT mechanism in real-life scenarios. Every correct character written in the correct position was counted as one point; for example, if the stimulus was “更元就” and the participant recalled it as “○元○,” they were given one point. Thus, the scores for this task ranged from 0 to 56. The choice to score by character (including whether it was in the correct position) rather than by word was made to ensure that the task effectively reflected the encoding features of non-sense processing of stimuli when processing information with MIBMT. In simple terms, if the stimulus was non-meaningfully processed, then even if the stimulus was presented as a non-word, each character that made up the non-word should have been processed individually during encoding; therefore, the character was the minimum retrieval unit for the task.

### Far-Transfer Tasks

#### Arithmetic Skills Task

Considering that participants included adolescents aged 10–16 years, to avoid both ceiling and floor effects, we referred to Huang’s (2018) method of constructing the task, using only addition and subtraction questions. We used six questions each on addition without regrouping (e.g., 33 + 62 = ?), addition with regrouping (e.g., 19 + 23 = ?), adding regrouping twice (e.g., 47 + 85 = ?), subtraction without regrouping (e.g., 97 – 42 = ?), subtraction with regrouping (e.g., 31 – 14 = ?), and subtraction with regrouping twice (e.g., 123 – 89 = ?) for 36 questions. No number greater than 1000 appeared during the calculation, and the numbers used were integers. After the question appeared on the screen, the participant used the keyboard to enter the answer in the dialog box and submit it; if no answer was submitted within 30 s, the system automatically displayed the next question, with a 1500 ms pause between questions. Participants did not receive feedback on the correctness of their answers during the task. The accuracy and response time (RT) were calculated separately.

#### Mental Rotation Task

This study used stimuli as described in Ganis and Kievit (2015). In a mental rotation task, the greater the angle of rotation, the longer the reaction time required (Estes, 2008). Therefore, we assigned the number of stimuli for each angle equally, wherein five groups were the same and five groups different at 0°, 50°, 100°, and 150°, resulting in 40 stimuli. Each set of stimuli was presented on the screen, and the participant had to use the keyboard (“O” for the same stimulus; “X” for a different stimulus) to make a judgment within 9000 ms; otherwise, the system would automatically display the next question. A blank screen was shown for 1500 ms between the questions. Participants did not receive feedback on whether their answers were correct during the task. The accuracy and RTs were calculated separately.

### Questionnaire: Post-training Evaluation

For the participants in the experimental group who completed the entire test, the questionnaire was evaluated on a five-point Likert scale: *used 0 times per week, used two times per week or less, used three to four times per week, used five to six times per week*, and *used daily*, allowing users to rate their frequency of MIBMT use during the one-year post-training.

### Procedure

**FIGURE 1:**
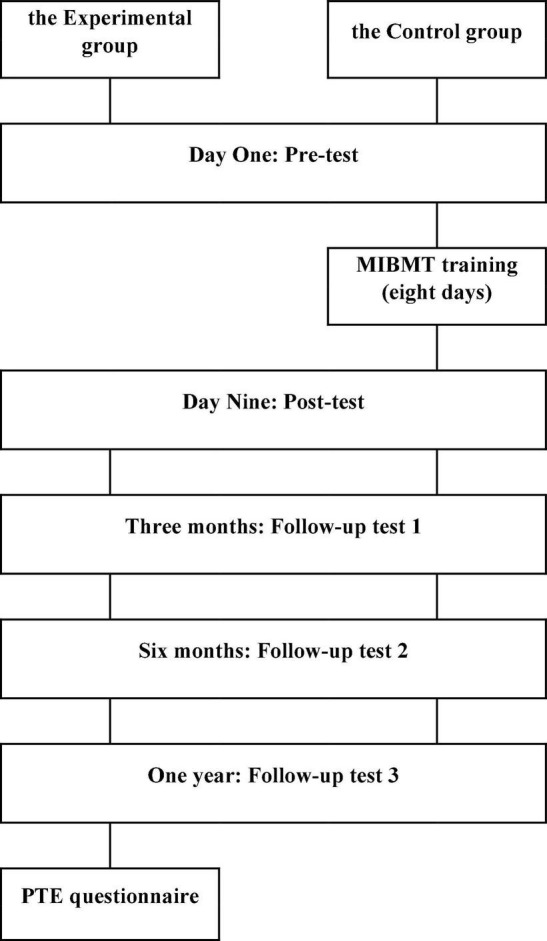
The procedure of experiment 1. MIBMT, mental-imagery-based mnemonic training; PTE, post-training evaluation.

The experimental group underwent eight days of MIBMT training (3 days of training, 3 days off, and 2 days of training), and the control group received no special training. All participants completed the pre-test on Day One of the study, the post-test on Day Nine, and three follow-up tests: 3 months, 6 months, and 1 year after the pre-test. Participants completed five tests in their computer classrooms. They were asked to complete the mental rotation and arithmetic skills tasks as quickly as possible while ensuring correct answers, whereas for the digit span and non-word recall tasks, participants were asked to recall items as accurately as possible without specific time limits. After all the five tests, which were spread across one year, were completed, the experimental group was asked to complete the Post-training Evaluation (PTE) questionnaire. For the procedure of Experiment 1, refer to Figure 1.

#### The Experimental Group

Mental-imagery-based mnemonic training began after the completion of the pre-test, and training sessions were held for 5 h per day (2.5 h in the morning and 2.5 h in the afternoon) (Table 4). Research suggests that spreading the training period out yields better training effects (Penner et al., 2012), as does the use of longer sessions (Schwaighofer et al., 2015). Jaeggi et al. (2008) also proposed that when more (vs. less) time is spent training, the improvement in Gf is greater. Our MIBMT study, with a total duration of 25 h over 8 days, took this into consideration.

**TABLE 4:** MIBMT training schedule.

	Day 1	Day 2	Day 3	Days 4–6	Day 7	Day 8
Morning (2 h 30 min)	Explanation of MIBMT (30 min)	Mindfulness practice (20 min)	Mindfulness practice (20 min)	Trying on applying MIBMT in daily lives	Providing feedback (20 min)	Mindfulness practice (20 min)
	Mindfulness practice (20 min)				Mindfulness practice (20 min)	
	MI-based memory training: meaningless characters (1 h 40 min: including the rest time)	MI-based memory training: Chinese phrases (2 h 10 min: including the rest time)	MI-based memory training: Chinese phrases (2 h 10 min: including the rest time)		MI-based memory training: Chinese and English phrases (1 h 50 min: including the rest time)	MI-based memory training: Chinese and English phrases (2 h 10 min: including the rest time)
Afternoon (2 h 30 min)	MI-based memory training: meaningless characters (2 h 30 min: including the rest time)	MI-based memory training: Chinese phrases (2 h 30 min: including the rest time)	MI-based memory training: Chinese and English phrases (2 h 15 min: including the rest time)		MI-based memory training: Chinese and English phrases (2 h 30 min: including the rest time)	MI-based memory training: Chinese and English phrases (2 h 30 min: including the rest time)
			Summarize the progress of the training (15 min)			

*MIBMT, mental-imagery-based mnemonic training; MI, mental imagery.*

The morning of Day One began with a half-hour explanation of MIBMT training. After the instructions were completed, a 20-min mindfulness practice was performed (only in the morning of each training day; no mindfulness practice was scheduled in the afternoon). In addition to MIBMT training instruction and mindfulness practice, time was spent on MI-based memory training. During the MI-based memory training, participants were asked to focus on the external characteristics of the target stimulus while encoding it and create the corresponding imagery personally. For example, when memorizing the number “6,” the participant should maintain the number “6” in mind, then define its color (e.g., red, yellow; participants who had difficulty building imagery on their own would be given a direct suggestion such as, “How about defining the image of 6 as yellow?” if necessary), tactile attributes (e.g., soft, rough), dimension (e.g., 2D, 3D), size (e.g., small, large), and other aspects to enhance the vividness of the imagery of “6.” MIBMT, unlike previous mnemonics, does not require the user to associate stimuli with other objects, such as animals or places, to promote recalling performance through associative means (e.g., association with spatial information, storytelling); rather, it creates individual imagery for each stimulus and reinforces their multi-sensory (but visually dominant) imagery traits.

The initial days consisted of the trainer’s reading of the stimulus to participants, with each set of stimuli consisting of seven meaningless characters (including *kanji*, numbers, letters, and shapes). The character contents are listed in Table 5. When reading each set of stimuli, the trainer read only one character at a time, with a pause between characters, ensuring that participants finished creating the relevant MI image before reading the next character. After participants had completed encoding each character in a set of stimuli using the MIBMT method, they were asked to repeat it in reverse order and then in the original order. After participants completed recalling the first set of stimuli, they encoded the next set of stimuli in the same way. However, during this training, participants were also required to recall the vertical order between groups of stimuli, for example, when asked about the third vertical column, participants had to recall the stimuli as they were presented from top to bottom (i.e., 看花的石 4 T) or from bottom to top (T 4 石的花看). In general, participants were required to grasp the location of each stimulus in Table 5 concerning the overall spatial relationship. The number of stimulation combinations presented depended on participants’ learning speed and capacity, and it might not have been possible for participants to recall all six stimulation groups on training day one.

**TABLE 5:** Meaningless character combinations used on day one.

1st combination	6	8	看	✩	△	A	1
2nd combination	E	3	花	□	育	0	本
3rd combination	5	D	的	+	8	Y	山
4th combination	年	D	石	5	○	2	×
5th combination	S	林	4	唱	B	回	7
6th combination	K	◇	T	8	明	U	久

On Day Two, the stimulus was replaced by simple Chinese phrases (about 10 Chinese characters in length), and participants were asked to memorize and recall them in the same way. The difference was that the trainer did not stop after each character while reading the sentence but read the entire sentence at a slower pace and read each sentence one to three times depending on participants’ capacity. Immediately after the trainer finished reading the stimuli, participants repeated them in reverse order and then in the original order (e.g., auditory presentation: “I think English is very difficult”; participant recalling: “difficult very is English think I”). Memorization of the Chinese stimuli only required participants to recall backwards or forwards and did not require grasping the top-down spatial relationship between sentences. After participants became relatively proficient in this method, sentence length was gradually increased and visual stimuli were introduced (i.e., participants were asked to read simple sentences and construct MI images, and then repeat them in the same way). This training did not require participants to get every character correct, but rather to note whether the content of their backward and forward recall was consistent even in the case of repeating errors (e.g., missing the same word). If it was too inconsistent—for example, when there were more than five inconsistencies between forward and backward recall—participants were reminded of that inconsistency in subsequent training attempts on the same stimuli because the stark difference between the content of backward and forward recall might be because of the participants’ high dependence on semantic processing.

On the afternoon of training Day Three, English stimuli were presented in the same way as the Chinese stimuli. The stimuli did not contain Chinese characters or English words that were beyond the range of the visual or auditory understanding of participants. Stimuli came directly from the later, unlearned portions of participants’ own Chinese or English textbooks. At the end of Day Three, 15 min were taken to summarize the progress of the training, and participants were asked to apply this method for what they needed to remember in their daily lives over the next 3 days.

At the beginning of training Day Four, participants were asked to provide feedback about what aspects of their life and studies incorporated MIBMT during the off days, how well they thought they had applied it, and whether they had any questions. The rest of Days Four and Five involved Chinese and English auditory and visual stimulus material, and stimuli were alternately presented, requiring participants to memorize and recall them using MIBMT. The difficulty of the stimuli, determined by the number of Chinese characters or English words used in a span, gradually increased according to participants’ performance. Chinese characters are not equivalent to English words or letters but are more like a combination of both; depending on the type of Chinese character, its meaning is sometimes equivalent to a word in English and sometimes only equivalent to a letter. Therefore, we adjusted the number of Chinese characters to control stimulus difficulty; stimuli that included more Chinese characters were considered more difficult. By contrast, the difficulty of the English stimuli was controlled by the number of words.

At the end of Day Eight, participants were asked to continue using MIBMT as actively as possible in their daily lives, but there was no special supervision post-training.

#### The Control Group

Before the experiment began, the control group was informed that this study tracked the development of memory, arithmetic, and spatial abilities in children and adolescents over a one-year period. Controls were not provided any information about MIBMT. They were only asked to go to the school’s computer room during the prescribed period to complete five separate tests to the best of their ability. After the five tests were completed, the purpose of this experiment was explained again to the control group.

### Data Analyses

Statistical analysis was performed with SPSS 25.0 (IBM; Armonk, NY, United States). We divided the experimental group into high-use (*n* = 10; five boys, five girls) and low-use (*n* = 8; six boys, two girls) groups according to the PTE score based on the median split technique (Rosen et al., 2003) and compared the changes in task performance of each group. The control group was used as the baseline. To reduce the possible underestimation of statistical power in between-groups comparisons in multiple time points, a 3 (high vs. low vs. control group) × 2 (pre-test and post-test; post-test and three-month follow-up test; post-test and 6-month follow-up test; post-test and 1-year follow-up test) analysis of variance (repeated measures ANOVA) was conducted to assess differences in the effect of the potential factor between the high, low, and control groups (Karbach et al., 2015). Furthermore, for significant interaction effects, we used the Ryan-Einot-Gabriel-Welsh F method as post-hoc analyses. Significance was set at *p* < 0.05, and *p* < 0.001 was considered highly significant. Additionally, we performed a post-hoc power analysis using G*Power (Faul et al., 2007) for 3 × 2 repeated measures ANOVA, and the effect size used in the calculation was partial eta-squared.

Single-task effect sizes were measured using Cohen’s *d*. The follow-up effect sizes were calculated in the same way as for the single-task effect size, using follow-up and pre-test results (Sala and Gobet, 2017a). Cohen’s *d* effect size was defined as small for *d* = 0.2, medium for *d* = 0.5, large for *d* = 0.8, and very large for *d* = 1.2 (Sawilowsky, 2009). That is, the larger the value, the greater the training effect.

### Results^1^

The means and standard deviations for each task are listed in Table 6. Group-occasion interactions and main effects for each group and occasion, as well as the effect size for each task, are listed in Tables 7–9.

**TABLE 6:** Means (standard deviations) of individual tasks in Experiment 1.

Means (*SD*)	Pre-test	Post-test	Three-month follow-up test	Six-month follow-up test	One-year follow-up test	Pre-test	Post-test	Three-month follow-up test	Six-month follow-up test	One-year follow-up test
**Near-transfer task**										

**Digit span (Score)**					**Non-word recall (Score)**					

High-use	9.20 (1.72)	10.40 (2.33)	9.65 (1.93)	10.85 (3.17)	9.30 (1.86)	16.10 (9.54)	23.70 (12.90)	28.90 (14.93)	31.20 (13.66)	31.60 (15.13)
Low-use	7.56 (0.32)	7.63 (1.19)	7.63 (0.64)	7.81 (0.53)	8.50 (1.28)	7.38 (5.37)	13.38 (7.44)	14.50 (8.64)	13.88 (7.40)	16.88 (11.85)
Control	7.44 (1.50)	8.06 (1.57)	8.69 (1.49)	7.99 (1.70)	8.56 (1.45)	10.88 (8.90)	5.13 (6.62)	5.75 (3.62)	7.13 (4.36)	8.50 (5.04)

**Far-transfer task**										

**Arithmetic skills (Accuracy)**					**Arithmetic skills (Response time)**					

High-use	0.87 (0.09)	0.88 (0.07)	0.90 (0.09)	0.89 (0.09)	0.91 (0.06)	372.50 (79.82)	356.31 (72.39)	318.42 (64.89)	332.82 (76.01)	339.36 (130.05)
Low-use	0.81 (0.07)	0.84 (0.11)	0.81 (0.18)	0.83 (0.11)	0.81 (0.15)	530.41 (131.98)	448.04 (57.42)	425.15 (93.97)	428.28 (86.32)	403.58 (54.69)
Control	0.79 (0.12)	0.82 (0.15)	0.82 (0.16)	0.81 (0.15)	0.81 (0.18)	456.30 (99.92)	416.71 (106.50)	397.55 (78.02)	351.25 (72.00)	382.92 (87.99)

**Mental rotation (Accuracy)**					**Mental rotation (Response time)**					

High-use	0.75 (0.13)	0.82 (0.14)	0.85 (0.14)	0.85 (0.18)	0.86 (0.12)	148.93 (49.14)	134.05 (44.94)	105.79 (24.56)	108.64 (33.21)	105.80 (33.34)
Low-use	0.84 (0.12)	0.88 (0.08)	0.86 (0.11)	0.86 (0.07)	0.87 (0.06)	145.99 (56.78)	132.41 (56.78)	123.24 (50.15)	123.92 (35.75)	120.14 (28.13)
Control	0.79 (0.10)	0.79 (0.18)	0.78 (0.17)	0.90 (0.06)	0.87 (0.08)	159.32 (45.53)	121.09 (54.24)	128.41 (42.37)	105.38 (23.87)	101.73 (25.19)

*SD, standard deviations.*

**TABLE 7:** ANOVA analysis and effect sizes of near transfer tasks in Experiment 1.

		Pre test - Post test	Post test - 3-month follow-up test	Post test - 6-month follow-up test	Post test - 1-year follow-up test
***Near transfer task***					

**Digit Span (Score)**					
**Effect Size**	High-use	**0.59 [−0.31, 1.48]**	−0.35 [−1.23, 0.53]	0.16 [−0.72, 1.04]	−**0.52 [**−**1.41, 0.37]**
	Low-use	0.07 [−0.91, 1.05]	0.00 [−0.98, 0.98]	0.20 [−0.78, 1.19]	**0.71 [**−**0.31, 1.72]**
	Control	0.41 [−0.58, 1.40]	0.41 [−0.58, 1.40]	−0.05 [−1.03, 0.93]	0.33 [−0.66, 1.32]

**Repeated Measures ANOVA**					

Main Effect	Occasion	***F* (1, 23) = 4.618, *p* = 0.042, ŋp^2^ = 0.167**	*F* (1, 23) = 0.020, *p* = 0.888, ŋp^2^ = 0.001	*F* (1, 23) = 0.153, *p* = 0.700, ŋp^2^ = 0.007	*F* (1, 23) = 0.048, *p* = 0.829, ŋp^2^ = 0.002
	Group	***F* (2, 23) = 6.866, *p* = 0.005, ŋp^2^ = 0.374**	***F* (2, 23) = 6.176, *p* = 0.007, ŋp^2^ = 0.349**	***F* (2, 23) = 8.957, *p* = 0.001, ŋp^2^ = 0.438**	***F* (2, 23) = 4.890, *p* = 0.017, ŋp^2^ = 0.298**
Group × Occasion		*F* (2, 23) = 1.311, *p* = 0.289, ŋp^2^ = 0.102	*F* (2, 23) = 1.919, *p* = 0.170, ŋp^2^ = 0.143	*F* (2, 23) = 0.104, *p* = 0.902, ŋp^2^ = 0.009	*F* (2, 23) = 2.231, *p* = 0.130, ŋp^2^ = 0.162
**Post hoc power**		0.829	0.946	0.118	0.971
**Nonwords Recall (Score)**					
**Effect Size**	High-use	**0.67 [**−**0.23, 1.57]**	**0.37 [**−**0.51, 1.26]**	**0.56 [**−**0.33, 1.46]**	**0.56 [**−**0.33, 1.46]**
	Low-use	**0.92 [**−**0.11, 1.96]**	**0.14 [**−**0.84, 1.12]**	**0.07 [**−**0.91, 1.05]**	**0.35 [**−**0.64, 1.34]**
	Control	−**0.73 [**−**1.75, 0.28]**	**0.12 [**−**0.86, 1.10]**	**0.36 [**−**0.63, 1.35]**	0.57 [−0.43, 1.58]

**Repeated Measures ANOVA**					

Main Effect	Occasion	***F* (1, 23) = 4.644, *p* = 0.042, ŋp^2^ = 0.168**	*F* (1, 23) = 1.779, *p* = 0.195, ŋp^2^ = 0.072	***F* (1, 23) = 4.954, *p* = 0.036, ŋp^2^ = 0.177**	***F* (1, 23) = 5.453, *p* = 0.029, ŋp^2^ = 0.192**
	Group	***F* (2, 23) = 5.030, *p* = 0.015, ŋp^2^ = 0.304**	***F* (2, 23) = 11.663, *p* = 0.000, ŋp^2^ = 0.504**	***F* (2, 23) = 13.126, *p* = 0.000, ŋp^2^ = 0.533**	***F* (2, 23) = 11.160, *p* = 0.000, ŋp^2^ = 0.492**
Group × Occasion		***F* (2, 23) = 11.819, *p* = 0.000, ŋp^2^ = 0.507**	*F* (2, 23) = 0.748, *p* = 0.484, ŋp^2^ = 0.061	*F* (2, 23) = 2.162, *p* = 0.138, ŋp^2^ = 0.158	*F* (2, 23) = 0.536, *p* = 0.592, ŋp^2^ = 0.045
***Post hoc* power**		1.000	0.578	0.967	0.443

*Cohen’s d-values above 0.5 (medium effect size) and p-values less than 0.05 (significant) are bold values.*

Regarding the near-transfer tasks, Table 7 shows no intentional interaction between group and occasion in the digit span task either between the pre- and post-test, or between the post-test and the individual follow-up trials (3 months, 6 months, and 1 year later). A simple main effect of group, however, was observed in the individual ANOVA analyses. In the post-hoc analysis, intentional differences were consistently observed between the high-use and low-use groups, and between the high-use and control groups, while there were no intentional differences between the low-use and control groups (Pre-test – Post-test: High-use – Low-use: *t* = 3.259, *p* = 0.003, High-use – Control: *t* = 3.028, *p* = 0.006, Low-use – Control: *t* = 0.219, *p* = 0.828; Post-test – 3-month follow-up test: High-use – Low-use: *t* = 3.392, *p* = 0.003, High-use – Control: *t* = 2.332, *p* = 0.029, Low-use – Control: *t* = 1.006, *p* = .325; Post-test – 6-month follow-up test: High-use – Low-use: *t* = 3.782, *p* = 0.001, High-use – Control: *t* = 3.383, *p* = 0.003, Low-use – Control: *t* = 0.378, *p* = 0.709; Post-test – 1-year follow-up test: High-use – Low-use: *t* = 2.837, *p* = 0.009, High-use – Control: *t* = 2.440, *p* = 0.023, Low-use – Control: *t* = 0.376, *p* = 0.710). In addition, although a simple main effect of one occasion was also observed between the pre- and post-test, only the high-use group showed a moderate effect size of 0.59 for increase in performance, and the effect size kept on fluctuating in the post- and the three follow-up tests, indicating that the WM-related task performance was not stable. The fluctuation in effect size was also seen in the control group. At the same time, although the effect size of the low-use group kept on increasing, this increase did not lead to a meaningful difference being observed between the low-use group and the control group. These data may indicate that there was an inconsistency in the baseline between the high-use group and the other two groups from the beginning, and that MIBMT and its frequency of use did not have a significant effect on the WM ability of the users.

As for the non-word recall task, a significant group × occasion interaction was observed between the pre- and post-test (Table 7). Post-hoc analysis revealed that both the high- and low-use groups showed an intentional increase in performance in the post-test compared to the pre-test [High-use: *F*(1,23) = 13.060, *p* = 0.002; Low-use: *F*(1,23) = 8.140, *p* = 0.009], in contrast to the control group, which showed an intentional decrease [Control: *F*(1,23) = 7.476, *p* = 0.012]. There were no intentional differences between the groups’ performance in the pre-test [*F*(2,46) = 2.017, *p* = 0.146], whereas the post-test showed significant differences [*F*(2,46) = 9.062, *p* = 0.001] between the high- and low-use groups (*t* = 2.405, *p* = 0.020), and between the high-use and control groups (*t* = 4.326, *p* = 0.000), and a tendency for significant differences between the low-use and control groups (*t* = 1.823, *p* = 0.075). Between the post-test and the individual follow-up tests (3 months, 6 months, and 1 year later), the simple main effect of group remained, although the intentional group × occasion interaction was no longer observed. *Post hoc* analysis showed that the differences between the high- and low-use groups, and between the high-use and control groups were maintained (Post-test – 3-month follow-up test: High-use – Low-use: *t* = 2.824, *p* = 0.010, High-use – Control: *t* = 4.765, *p* = 0.000, Low-use – Control: *t* = 1.842, *p* = 0.078; Post-test – 6-month follow-up test: High-use – Low-use: *t* = 3.239, *p* = 0.004, High-use – Control: *t* = 4.996, *p* = 0.000, Low-use – Control: *t* = 1.667, *p* = 0.109; Post-test – One-year follow-up test: High-use - Low-use: *t* = 2.797, *p* = 0.010, High-use – Control: *t* = 4.654, *p* = 0.000, Low-use – Control: *t* = 1.761, *p* = 0.091). The effect size also showed that the gains in non-word recall tasks in the high- and low-use groups in the post-test compared to the pre-test were maintained in the subsequent follow-up tests. This suggests that the continuous use of MIBMT in daily life had a positive effect on improving LTM capacity, as users who tended to actively use MIBMT showed better LTM performance post-training.

Regarding the arithmetic skills task, according to Table 8, both accuracy and RT of the task showed no significant effect of experimental group × occasion interaction. In the pre-test, the post-hoc analysis revealed that the high- and low-use groups showed an intentional between-group difference in RT (*t* = 3.347, *p* = 0.003). There was also an intentional tendency that appeared between the high-use and control groups (*t* = 1.933, *p* = 0.066), while there were no intentional differences between the low-use and control groups (*t* = 1.341, *p* = 0.193). Referring to the effect sizes of the groups and Table 6, it seems that there is an inconsistency in the baseline of RT for arithmetic skills between the high-use group and the other two groups probably from pre-test onwards (Post-test – 3-month follow-up test: High-use – Low-use: *t* = 2.984, *p* = 0.007, High-use – Control: *t* = 2.098, *p* = 0.047, Low-use – Control: *t* = 0.841, *p* = 0.409; Post-test – 6-month follow-up test: High-use – Low-use: *t* = 2.726, *p* = 0.012, High-use – Control: *t* = 1.148, *p* = 0.263, Low-use – Control: *t* = 1.497, *p* = 0.148). No evidence was found that MIBMT and its frequency of use improved users’ performance in arithmetic skills.

**TABLE 8:** ANOVA analysis and effect sizes of far transfer arithmetic skills task in Experiment 1.

		Pre test - Post test	Post test - 3-month follow-up test	Post test - 6-month follow-up test	Post test - 1-year follow-up test
***Far transfer task***					

**Arithmetic skills (Accuracy)**					
**Effect Size**	High-use	0.07 [−0.81, 0.94]	0.31 [−0.57, 1.19]	0.17 [−0.70, 1.05]	0.46 [−0.43, 1.35]
Low-use	0.34 [−0.65, 1.33]	−0.25 [−1.23, 0.74]	−0.11 [−1.09, 0.87]	−0.25 [−1.23, 0.74]	
Control	0.23 [−0.76, 1.21]	0.03 [−0.95, 1.01]	−0.04 [−1.02, 0.94]	−0.04 [−1.02, 0.94]	

**Repeated Measures ANOVA**					

Main Effect	Occasion	*F* (1, 23) = 2.064, *p* = 0.164, ŋp^2^ = 0.082	*F* (1, 23) = 0.007, *p* = 0.933, ŋp^2^ = 0.000	*F* (1, 23) = 0.009, *p* = 0.924, ŋp^2^ = 0.000	*F* (1, 23) = 0.011, *p* = 0.918, ŋp^2^ = 0.000
	Group	*F* (2, 23) = 1.228, *p* = 0.311, ŋp^2^ = 0.097	*F* (2, 23) = 1.126, *p* = 0.341, ŋp^2^ = 0.089	*F* (2, 23) = 0.935, *p* = 0.407, ŋp^2^ = 0.075	*F* (2, 23) = 1.457, *p* = 0.254, ŋp^2^ = 0.112
	Group × Occasion	*F* (2, 23) = 0.323, *p* = 0.727, ŋp^2^ = 0.027	*F* (2, 23) = 0.429, *p* = 0.656, ŋp^2^ = 0.036	*F* (2, 23) = 0.262, *p* = 0.772, ŋp^2^ = 0.022	*F* (2, 23) = 0.448, *p* = 0.645, ŋp^2^ = 0.037
***Post hoc* power**		0.277	0.360	0.231	0.370

**Arithmetic skills (Response time)**					

**Effect Size**	High-use	0.21 [−0.67, 1.09]	**0.55 [**−**0.34, 1.45]**	0.32 [−0.57, 1.20]	0.16 [−0.72, 1.04]
	Low-use	0.81 [−0.22, 1.83]	0.29 [−0.69, 1.28]	0.27 [−0.72, 1.25]	**0.79 [**−**0.23, 1.82]**
	Control	0.38 [−0.61, 1.37]	0.21 [−0.78, 1.19]	**0.72 [**−**0.30, 1.74]**	0.35 [−0.64, 1.33]

**Repeated Measures ANOVA**					

Main Effect	Occasion	***F* (1, 23) = 5.479, *p* = 0.028, ŋp^2^ = 0.192**	*F* (1, 23) = 3.189, *p* = 0.087, ŋp^2^ = 0.122	***F* (1, 23) = 7.910, *p* = 0.010, ŋp^2^ = 0.256**	***F* (1, 23) = 5.598, *p* = 0.027, ŋp^2^ = 0.196**
	Group	***F* (2, 23) = 5.724, *p* = 0.010, ŋp^2^ = 0.332**	***F* (2, 23) = 4.823, *p* = 0.018, ŋp^2^ = 0.295**	***F* (2, 23) = 3.718, *p* = 0.040, ŋp^2^ = 0.244**	*F* (2, 23) = 2.038, *p* = 0.153, ŋp^2^ = 0.151
	Group × Occasion	*F* (2, 23) = 0.988, *p* = 0.388, ŋp^2^ = 0.079	*F* (2, 23) = 0.158, *p* = 0.855, ŋp^2^ = 0.014	*F* (2, 23) = 1.242, *p* = 0.307, ŋp^2^ = 0.097	*F* (2, 23) = 0.376, *p* = 0.691, ŋp^2^ = 0.032
***Post hoc* power**		0.707	0.160	0.806	0.323

*Cohen’s d-values above 0.5 (medium effect size) and p-values less than 0.05 (significant) are bold values.*

Concerning the mental rotation task, Table 9 shows that neither the accuracy of the task nor RT showed significant effects for group and occasion. In combination with the effect sizes of individual groups and Table 6, this suggests that MIBMT had no effect on users’ performance in the mental rotation task.

**TABLE 9:** ANOVA analysis and effect sizes of far transfer mental rotation task in Experiment 1.

		Pre test - Post test	Post test - 3-month follow-up test	Post test - 6-month follow-up test	Post test - 1-year follow-up test
***Far transfer task***					

**Mental rotation (Accuracy)**					
**Effect Size**	High-use	**0.54 [**−**0.36, 1.43]**	0.20 [−0.68, 1.08]	0.19 [−0.69, 1.07]	0.27 [ = 0.61, 1.15]
	Low-use	0.37 [−0.62, 1.36]	−0.23 [−1.21, 0.76]	−0.28 [−1.27, 0.70]	−0.13 [−1.11, −0.86]
	Control	−0.02 [−1.00, 0.96]	−0.06 [−1.04, 0.92]	**0.84 [**−**0.19, 1.87]**	**0.55 [**−**0.45, 1.55]**

**Repeated Measures ANOVA**					

Main Effect	Occasion	*F* (1, 23) = 2.284, *p* = 0.144, ŋp^2^ = 0.090	*F* (1, 23) = .006, *p* = 0.937, ŋp^2^ = 0.000	*F* (1, 23) = 3.657, *p* = 0.068, ŋp^2^ = 0.137	*F* (1, 23) = 2.757, *p* = 0.110, ŋp^2^ = 0.107
	Group	*F* (2, 23) = 1.205, *p* = 0.318, ŋp^2^ = 0.095	*F* (2, 23) = 0.881, *p* = 0.428, ŋp^2^ = 0.071	*F* (2, 23) = 0.200, *p* = 0.820, ŋp^2^ = 0.017	*F* (2, 23) = 0.486, *p* = 0.621, ŋp^2^ = 0.041
	Group × Occasion	*F* (2, 23) = 0.878, *p* = 0.429, ŋp^2^ = 0.071	*F* (2, 23) = 0.827, *p* = 0.450, ŋp^2^ = 0.067	*F* (2, 23) = 3.234, *p* = 0.058, ŋp^2^ = 0.220	*F* (2, 23) = 1.372, *p* = 0.274, ŋp^2^ = 0.107
***Post hoc* power**		0.653	0.624	0.997	0.849

**Mental rotation (Response time)**					

**Effect Size**	High-use	0.32 [−0.57, 1.20]	**0.78 [**−**0.13, 1.69]**	**0.64 [**−**0.26, 1.54]**	**0.71 [**−**0.19, 1.62]**
	Low-use	0.24 [−0.74, 1.22]	0.17 [−0.81, 1.15]	0.18 [−0.80, 1.16]	0.27 [−0.71, 1.26]
	Control	**0.76 [**−**0.26, 1.78]**	0.15 [−0.83, 1.13]	−0.37 [−1.36, 0.61]	−0.46 [−1.45, 0.54]

**Repeated Measures ANOVA**					

Main Effect	Occasion	***F* (1, 23) = 9.856, *p* = 0.005, ŋp^2^ = 0.300**	*F* (1, 23) = 3.128, *p* = 0.090, ŋp^2^ = 0.120	*F* (1, 23) = 2.980, *p* = 0.098, ŋp^2^ = 0.115	***F* (1, 23) = 7.222, *p* = 0.013, ŋp^2^ = 0.239**
	Group	*F* (2, 23) = 0.005, *p* = 0.995, ŋp^2^ = 0.000	*F* (2, 23) = 0.076, *p* = 0.927, ŋp^2^ = 0.007	*F* (2, 23) = 0.360, *p* = 0.701, ŋp^2^ = 0.030	*F* (2, 23) = 0.315, *p* = 0.733, ŋp^2^ = 0.027
	Group × Occasion	*F* (2, 23) = 1.232, *p* = 0.310, ŋp^2^ = 0.097	*F* (2, 23) = 3.283, *p* = 0.056, ŋp^2^ = 0.230	*F* (2, 23) = 0.275, *p* = 0.762, ŋp^2^ = 0.023	*F* (2, 23) = 0.407, *p* = 0.671, ŋp^2^ = 0.034
***Post hoc* power**		0.102	0.998	0.240	0.342

*Cohen’s d-values above 0.5 (medium effect size) and p-values less than 0.05 (significant) are bold values.*

### Discussion

We found that MIBMT improved participants’ LTM task performance and had a maintenance effect for at least 1 year. Further, the active use of MIBMT in daily life may reflect users’ acceptance of the training, which may in turn affect their motivation during the training. The results showed that participants who could actively use MIBMT later performed better on post-tests related to LTM capabilities, with a further long-term maintenance effect. There was no evidence that MIBMT improved the performance of WM-related short-term tasks. The difference in the performance of the groups on WM and LTM tasks may be because MIBMT helped improve the efficiency and quality of encoding while dealing with LTM-related tasks rather than improving task performance by increasing the storage capacity of WM (Carretti et al., 2007). Considering extant WM models, these models have two aspects in common: the limited capacity of WM and cognitive control capacity (Baddeley, 2003; Unsworth and Engle, 2007; Schwaighofer et al., 2015). While the digit span task focused only on the limited capacity of WM, to further explore the relationship between MIBMT, WM, and LTM, it was necessary to use a Stroop task in Experiment 2 that could measure participants’ capacity for cognitive control (e.g., Pugin et al., 2014; Karbach et al., 2015).

In the case of far transfer, the results suggest that MIBMT had no effect on users’ arithmetic and spatial abilities. The main goal of cognitive training is to improve or maintain the Gf of users (Tsubomi et al., 2019), and Gf includes the arithmetic, spatial, and reasoning abilities (Jaeggi et al., 2008). Morrison and Chein (2011) noted that the SPM task developed by Raven (1936) as a non-verbal Gf test is an appropriate tool for assessing the far-transfer effects of cognitive training. Therefore, to further clarify the effectiveness of MIBMT as a cognitive training, it was necessary to use the SPM task in Experiment 2.

Experiment 1 examined the effectiveness of MIBMT in LTM and the possibility of maintaining this effect. The frequency of daily use may be a factor that influences the effectiveness of MIBMT. Further, Experiment 1 had four limitations: first, for WM capacity, Experiment 1 tested only the storage capacity of the participants and not other associated capacities, such as the distribution or control of attention in the WM system; second, we did not utilize some mainstream testing methods such as the SPM task for exploring Gf (reasoning ability) improvement in far transfer. Third, inconsistencies in the baseline between groups emerged in the WM and arithmetic tasks, which may be a limitation of the too small sample size. Fourth, although the goal of MIBMT is to allow users to easily and consistently apply it in their daily lives, the daily use of MIBMT by participants was measured using only a single Likert scale. To further investigate the usefulness of MIBMT, more surveys of participants’ use status are necessary.

## Experiment 2

### Objectives

Based on the results and limitations of Experiment 1, four modifications were made in Experiment 2: first, the digit span task was changed to a Stroop task to fully measure WM function; second, an SPM task was added to measure users’ Gf; third, the experimental participants were limited to sixth graders, and the experimental period was changed to 3 months to maximize sample size. Fourth, 3 months after the training, parents of all participants in the experimental group were asked to submit feedback on their children’s use status of MIBMT, academic performance, and daily life status during the three months (since the participants were elementary school students, we chose their parents as the reporter to ensure the objectivity and accuracy of the feedback). We conducted a contents analysis to investigate the impact of MIBMT on daily life. Experiment 2 is a revalidation of the results of Experiment 1 and an extension of the study in terms of both quantitative and qualitative analyses to further clarify the effectiveness and sustainability of MIBMT, as well as the impact of the frequency of daily use of MIBMT.

### Ethical Approval

The same as Experiment 1.

### Participants

From December 2020 to January 2021, 54 sixth graders (all 12 years old; 28 boys, 26 girls) from an elementary school in Yangzhou, Jiangsu Province, China, participated in the study. The experimental and control groups comprised 27 participants each (17 boys, 10 girls and 16 girls, 11 boys, respectively). Participants were recruited and grouped, and they participated in the same manner as in Experiment 1. Children who participated in Experiment 1 were not included in Experiment 2.

### Materials

All tasks were originally designed by the researcher and ran on E-prime 2.0. The tasks of each test ( pre-, post-, and follow-up) used different stimulus questions derived under the same rule/source, which means that all participants completed the same tasks in the three tests, but the questions differed between tasks.

### Near-Transfer Tasks

#### Stroop Task

This task was designed based on the work of Zysset et al. (2001). Each stimulus was presented on the screen, and the participant had to use the keyboard (“O” for the same stimulus; “X” for a different stimulus) to make a judgment within 4000 ms; otherwise, the system would automatically display the next question. We assigned the number of stimuli of “same” and “different” equally in the task, and no blanks were placed between stimuli. The task session was limited to 4500 ms to balance accuracy and RT. A correct answer was scored as one point and an incorrect answer as minus one point (Scarpina and Tagini, 2017). Participants did not receive feedback on whether their answers were correct during the task.

#### Non-word Recall Task

The same task as in Experiment 1 was used.

### Far-Transfer tasks

#### Arithmetic Skills Task

The same task as in Experiment 1 was used.

#### Mental Rotation Task

The same task as in Experiment 1 was used.

#### Raven’s Standard Progressive Matrices Task

We used the Chinese version of the SPM task (60 questions), revised by Zhang and Wang (1989). The first three questions were used as practice examples, and the remaining 57 questions were divided equally into three sets—A, B, and C—rotating in multiples of three by question number; each set (containing 19 questions) was used as a pre-, post-, and follow-up test (Wang et al., 2014). After the question appeared on the screen, the participant used the keyboard to enter the answer in the dialog box and submit it. No time limit was set for each question, but the time limit for the entire SPM task was 10 min. A correct answer was counted as one point and an incorrect answer as 0 points (Studer-Luethi et al., 2016). Score and RT were calculated separately.

### Questionnaire: Post-training Evaluation

For the participants in the experimental group who completed the whole test, the questionnaire was evaluated on a five-point Likert scale: *used 0 times per week, used two times per week or less, used three to four times per week, used five to six times per week*, and *used daily*, allowing users to rate their frequency of MIBMT use at three months post-training.

### Procedure

The experimental group underwent eight days of MIBMT training (3 days of training, 3 days off, and 2 days of training), and the control group received no special training. All participants completed the pre-test on Day One of the study, the post-test on Day Nine, and the follow-up test three months after the pre-test. Participants completed the three tests in their computer classrooms. They were instructed to complete the Stroop task, arithmetic skills task, mental rotation task, and SPM task as quickly as possible while ensuring correct answers; for the non-word recall task, they were asked to recall items as accurately as possible without specific time limits.

The experimental group received the same eight-day MIBMT training as in Experiment 1. After all the three tests, which spread across 3 months, were completed, the experimental group was asked to complete the PTE questionnaire. Parents were asked to submit feedback on their child’s use of MIBMT and their academic performance during the three months. The control group was informed before the experiment that this study tracked the development of memory, arithmetic, and spatial abilities in children and adolescents over a three-month period. Controls were not provided any information about MIBMT. They were only asked to go to the school’s computer room during the prescribed period to complete three separate tests to the best of their ability. After the three tests were completed, the purpose of this experiment was explained again to the control group.

### Quantitative Analysis

#### Data Analyses

Statistical analysis was performed with SPSS 25.0 (IBM; Armonk, NY, United States). We divided the experimental group into high-use (*n* = 11; 6 boys, 5 girls) and low-use (*n* = 16; 11 boys, 5 girls) groups according to the PTE score based on the median split technique (Rosen et al., 2003) and compared the changes in task performance of each group. The control group was used as the baseline. Repeated measures ANOVA and *post hoc* analyses for significant interaction effects, *post hoc* power analysis, and single-task effect sizes were consistent with those in Experiment 1.

#### Results

The means and standard deviations for each task are listed in Table 10. Group × occasion interactions and main effects for each group and occasion, as well as the effect size for each task, are listed in Tables 11–14.

**TABLE 10:** Means (standard deviations) of near transfer tasks in Experiment 2.

Means (*SD*)	Pre-test	Post-test	Three-month follow-up test	Pre-test	Post-test	Three-month follow-up test
**Near-transfer task**						

**Stroop (Score)**			**Non-word recall (Score)**			
High-use	13.64 (4.13)	17.45 (3.50)	19.82 (2.48)	13.00 (7.80)	25.91 (15.31)	29.91 (17.54)
Low-use	12.06 (6.31)	15.81 (6.68)	18.88 (6.44)	8.63 (5.99)	16.88 (8.85)	21.63 (10.51)
Control	13.11 (5.89)	14.81 (5.23)	17.78 (5.14)	14.41 (5.94)	10.59 (5.77)	9.93 (5.15)

**Far-transfer task**						

**Arithmetic skills (Accuracy)**			**Arithmetic skills (Response time)**			

High-use	0.90 (0.06)	0.92 (0.06)	0.91 (0.03)	426.52 (80.68)	398.34 (86.18)	386.29 (84.81)
Low-use	0.85 (0.15)	0.84 (0.17)	0.83 (0.16)	414.96 (109.10)	364.45 (115.46)	351.35 (107.54)
Control	0.80 (0.14)	0.84 (0.08)	0.87 (0.08)	369.64 (86.45)	373.72 (88.04)	326.47 (78.89)

**Mental rotation (Accuracy)**			**Mental rotation (Response time)**			

High-use	0.77 (0.13)	0.83 (0.10)	0.90 (0.12)	160.39 (44.56)	124.70 (27.26)	115.87 (32.01)
Low-use	0.82 (0.13)	0.88 (0.07)	0.84 (0.14)	134.86 (24.52)	117.78 (25.16)	106.36 (30.68)
Control	0.81 (0.13)	0.87 (0.10)	0.90 (0.07)	132.60 (30.84)	115.62 (23.54)	106.59 (20.65)

**SPM (Score)**			**SPM (Response time)**			

High-use	14.64 (2.84)	14.09 (1.97)	14.27 (2.41)	373.03 (153.04)	244.09 (102.02)	227.98 (107.73)
Low-use	14.69 (2.36)	13.38 (3.46)	13.56 (2.42)	364.07 (145.82)	287.71 (141.48)	266.82 (169.44)
Control	13.30 (1.96)	12.85 (1.83)	11.89 (2.89)	232.92 (79.95)	241.27 (67.42)	217.32 (63.75)

*SD, standard deviations; SPM, Raven’s standard progressive matrices.*

**TABLE 11:** ANOVA analysis and effect sizes of individual tasks in Experiment 2.

		Pre test – Post test	Post test – Three-month follow-up test
***Near transfer task***			

**Stroop (Score)**			

**Effect Size**	High-use	**1.00 [0.11, 1.89]**	**0.78 [–0.09, 1.65]**
	Low-use	**0.58 [–0.13, 1.29]**	0.47 [–0.24, 1.17]
	Control	0.31 [–0.23, 0.84]	**0.57 [0.03, 1.12]**

**Repeated Measures ANOVA**			

Main Effect	Occasion	***F*(1,51) = 15.918, *p* = 0.000, ${ŋ}_{\text{p}}^{2}$ = 0.238**	***F*(1,51) = 15.169, *p* = 0.000, ${ŋ}_{\text{p}}^{2}$ = 0.229**
	Group	*F*(2,51) = 0.463, *p* = 0.632, ${ŋ}_{\text{p}}^{2}$ = 0.018	*F*(2,51) = 1.004, *p* = 0.373, ${ŋ}_{\text{p}}^{2}$ = 0.038
Group × Occasion		*F*(2,51) = 1.024, *p* = 0.366, ${ŋ}_{\text{p}}^{2}$ = 0.039	*F*(2,52) = 0.075, *p* = 0.928, ${ŋ}_{\text{p}}^{2}$ = 0.003
***Post hoc* power**		0.732	0.099

**Non-words Recall (Score)**			

**Effect Size**	High-use	**1.06 [0.16, 1.96]**	0.24 [–0.60, 1.08]
	Low-use	**1.09 [0.35, 1.84]**	0.49 [–0.21, 1.19]
	Control	**–0.65 [–1.20, –0.10]**	–0.12 [–0.66, 0.41]

**Repeated Measures ANOVA**			

Main Effect	Occasion	***F*(1,51) = 20.384, *p* = 0.000, ${ŋ}_{\text{p}}^{2}$ = 0.286**	***F*(1,51) = 5.589, *p* = 0.022, ${ŋ}_{\text{p}}^{2}$ = 0.099**
	Group	***F*(2,51) = 4.709, *p* = 0.013, ${ŋ}_{\text{p}}^{2}$ = 0.156**	***F*(2,51) = 16.115, *p* = 0.000, ${ŋ}_{\text{p}}^{2}$ = 0.387**
Group × Occasion		***F*(2,51) = 17.896, *p* = 0.000, ${ŋ}_{\text{p}}^{2}$ = 0.412**	*F*(2,51) = 2.902, *p* = 0.064, ${ŋ}_{\text{p}}^{2}$ = 0.102
***Post hoc* power**		1.000	0.994

*Cohen’s d-values above 0.5 (medium effect size) and p-values less than 0.05 (significant) are bold values.*

Regarding the near transfer to WM and LTM tasks (Table 11), no significant difference was observed in the WM-related Stroop task. Performances of all groups improved in the Stroop task. A significant group × occasion (pre-test, post-test) interaction was observed in the LTM task. *Post hoc* analysis revealed that both the high- and low-use groups showed intentional increases in performance in the post-test compared to the pre-test [High-use: *F*(1,51) = 33.876, *p* = 0.000; Low-use: *F*(1,51) = 13.836, *p* = 0.001; Control: *F*(1,51) = 2.958, *p* = 0.092]. There were no intentional differences observed in the performance of the groups in the pre-test [*F*(2,102) = 2.265, *p* = 0.109], while the post-test showed significant differences [*F*(2,102) = 14.768, *p* = 0.000] between the high- and low-use groups (*t* = 2.901, *p* = 0.005), the high-use and control groups (*t* = 5.385, *p* = 0.000), and the low-use and control groups (*t* = 2.504, *p* = 0.014). Although a group × occasion (post-test, 3-month follow-up test) intentional interaction was no longer observed, the group-only main effect remained. *Post hoc* analyses showed that the differences between the high- and low-use groups (*t* = 2.459, *p* = 0.017), the high-use and control groups (*t* = 5.489, *p* = 0.000), and the low-use and control groups were maintained (*t* = 3.170, *p* = 0.003). The effect size also showed that the gains in non-word recall tasks in the high- and low-use groups in the post-test compared to the pre-test were maintained in the subsequent follow-up test. This suggests that MIBMT had a positive effect on improving LTM performance, and that users who tended to use MIBMT more frequently showed better LTM performance post-training.

Regarding the arithmetic skills task, combined with Table 12, both accuracy and RT of the task showed no significant effect of experimental group × occasion interaction (pre-test, post-test; post-test, 3-month follow-up test), which indicates that the MIBMT may not improve users’ performance in arithmetic skills. Concerning the mental rotation task (Table 13), both accuracy and RT of the task showed no significant effect of group × occasion interaction (pre-test, post-test), but group × occasion interaction of task accuracy (post-test; 3-month follow-up test) showed a significant effect. *Post hoc* analysis showed that only the high-use group in follow-up test showed an intentional increase in performance compared to the post-test [High-use: *F*(1,51) = 5.747, *p* = 0.020; Low-use: *F*(1,51) = 1.965, *p* = 0.167; Control: *F*(1,51) = 1.225, *p* = 0.274]. Nevertheless, no significant differences in performance were observed between the high- and low-use groups, the high-use and control groups, and the low-use and control groups in either the post-test or the three-month follow-up test [Post-test: *F*(2,102) = 1.353, *p* = 0.263; 3-month follow-up test: *F*(2,102) = 1.538, *p* = 0.220]. Combined with the effect sizes and Table 10, the present results are not sufficient to suggest a positive effect of MIBMT on users’ performance on the mental rotation task.

**TABLE 12:** ANOVA analysis and effect sizes of far transfer arithmetic skills task in Experiment 2.

		Pre test - Post test	Post test - 3-month follow-up test
***Far transfer task***			

**Arithmetic skills (Accuracy)**			

**Effect Size**	High-use	0.20 [−0.64, 1.04]	−0.10 [−0.94, 0.74]
	Low-use	−0.08 [−0.77, 0.61]	−0.05 [−0.75, 0.64]
	Control	0.43 [−0.11, 0.97]	0.30 [−0.24, 0.84]

**Repeated Measures ANOVA**			

Main Effect	Occasion	*F* (1, 51) = 1.047, *p* = 0.311, ŋp^2^ = 0.020	*F* (1, 51) = 0.060, *p* = 0.808, ŋp^2^ = 0.001
	Group	*F* (2, 51) = 2.822, *p* = 0.069, ŋp^2^ = 0.100	*F* (2, 51) = 2.308, *p* = 0.110, ŋp^2^ = 0.083
Group × Occasion		*F* (2, 51) = 1.641, *p* = 0.204, ŋp^2^ = 0.060	*F* (2, 51) = 0.747, *p* = 0.479, ŋp^2^ = 0.028
***Post hoc* power**		0.908	0.578

**Arithmetic skills (Response time)**			

**Effect Size**	High-use	0.34 [−0.50, 1.18]	0.14 [−0.70, 0.98]
	Low-use	0.45 [−0.25, 1.15]	0.12 [−0.58, 0.81]
	Control	−0.05 [−0.58, 0.49]	0.57 [0.02, 1.11]

**Repeated Measures ANOVA**			

Main Effect	Occasion	***F* (1, 51) = 6.826, *p* = 0.012, ŋp^2^ = 0.118**	***F* (1, 51) = 9.364, *p* = 0.004, ŋp^2^ = 0.155**
	Group	*F* (2, 51) = 0.850, *p* = 0.433, ŋp^2^ = 0.032	*F* (2, 51) = 0.893, *p* = 0.416, ŋp^2^ = 0.034
Group × Occasion		*F* (2, 51) = 2.771, *p* = 0.072, ŋp^2^ = 0.125	*F* (2, 51) = 2.747, *p* = 0.074, ŋp^2^ = 0.097
***Post hoc* power**		0.999	0.991

*Cohen’s d-values above 0.5 (medium effect size) and p-values less than 0.05 (significant) are bold values.*

**TABLE 13:** ANOVA analysis and effect sizes of far transfer mental rotation task in Experiment 2.

		Pre test - Post test	Post test - 3-month follow-up test
***Far transfer task***			

**Mental rotation (Accuracy)**			

**Effect Size**	High-use	0.48 [−0.37, 1.33]	**0.63 [**−**0.23, 1.49]**
	Low-use	**0.58 [**−**0.13, 1.29]**	−0.36 [−1.06, 0.34]
	Control	0.44 [−0.10, 0.98]	0.36 [−0.18, 0.90]

**Repeated Measures ANOVA**			

Main Effect	Occasion	***F* (1, 51) = 10.300, *p* = 0.002, ŋp^2^ = 0.168**	*F* (1, 51) = 1.473, *p* = 0.230, ŋp^2^ = 0.028
	Group	*F* (2, 51) = 1.066, *p* = 0.352, ŋp^2^ = 0.040	*F* (2, 51) = 0.435, *p* = 0.650, ŋp^2^ = 0.017
Group × Occasion		*F* (2, 51) = 0.013, *p* = 0.987, ŋp^2^ = 0.001	***F* (2, 51) = 3.382, *p* = 0.042, ŋp^2^ = 0.117**
***Post hoc* power**		0.066	0.998

**Mental rotation (Response time)**			

**Effect Size**	High-use	**0.97 [0.08, 1.85]**	0.30 [−0.54, 1.14]
	Low-use	**0.69 [**−**0.03, 1.40]**	0.41 [−0.29, 1.11]
	Control	**0.62 [0.07, 1.17]**	0.41 [−0.13, 0.95]

**Repeated Measures ANOVA**			

Main Effect	Occasion	***F* (1, 51) = 25.856, *p* = 0.000, ŋp^2^ = 0.336**	***F* (1, 51) = 6.984, *p* = 0.011, ŋp^2^ = 0.120**
	Group	*F* (2, 51) = 2.360, *p* = 0.105, ŋp^2^ = 0.085	*F* (2, 51) = 0.700, *p* = 0.501, ŋp^2^ = 0.027
Group × Occasion		*F* (2, 51) = 1.545, *p* = 0.223, ŋp^2^ = 0.057	*F* (2, 51) = 0.053, *p* = 0.949, ŋp^2^ = 0.002
***Post hoc* power**		‘0.891	0.082

*Cohen’s d-values above 0.5 (medium effect size) and p-values less than 0.05 (significant) are bold values.*

For the SPM task (Table 14), no group × occasion interaction was observed in the task scores, while the mean RT of the task showed a significant interaction between group and occasion (pre-test; post-test). *Post hoc* analysis showed that both the high- and low-use groups showed an intentional increase in response speed in the post-test compared to the pre-test [High-use: *F*(1,51) = 28.543, *p* = 0.000; Low-use: *F*(1,51) = 10.011, *p* = 0.003; Control: *F*(1,51) = 0.120, *p* = 0.731]. A significant difference was observed between the high-use and control groups and the low-use and control groups in the pre-test [*F*(2,102) = 7.960, *p* = 0.001; High-use – Low-use: *t* = 0.207, *p* = 0.836, High-use – Control: *t* = 3.550, *p* = 0.001, Low-use – Control: *t* = 3.768, *p* = 0.000]; however, no intentional differences were observed in the performance of the groups in the post-test [*F*(2,102) = 0.877, *p* = 0.419]. Combined with the effect sizes and Table 10, these data may indicate that there was an inconsistency in the baseline for the SPM task between the experimental and control groups from the very beginning and that MIBMT and its frequency of use may not have had a significant effect on users’ performance in the SPM task.

**TABLE 14:** ANOVA analysis and effect sizes of far transfer SPM task in Experiment 2.

		Pre test - Post test	Post test - 3-month follow-up test
***Far transfer task***			

**SPM (Score)**			

**Effect Size**	High-use	−0.22 [−1.06, 0.62]	0.08 [−0.75, 0.92]
	Low-use	−0.44 [−1.15, 0.26]	0.06 [−0.63, 0.76]
	Control	−0.23 [−0.77, 0.30]	−0.40 [−0.94, 0.14]

**Repeated Measures ANOVA**			

Main Effect	Occasion	***F* (1, 51) = 4.190, *p* = 0.046, ŋp^2^ = 0.076**	*F* (1, 51) = 0.292, *p* = 0.591, ŋp^2^ = 0.006
	Group	*F* (2, 51) = 2.145, *p* = 0.127, ŋp^2^ = 0.078	*F* (2, 51) = 2.951, *p* = 0.061, ŋp^2^ = 0.104
Group × Occasion		*F* (2, 51) = 0.602, *p* = 0.551, ŋp^2^ = 0.023	*F* (2, 51) = 1.405, *p* = 0.255, ŋp^2^ = 0.052
***Post hoc* power**		0.486	0.858

**SPM (Response time)**			

**Effect Size**	High-use	**0.99 [0.10, 1.88]**	0.15 [−0.68, 0.99]
	Low-use	**0.53 [**−**0.17, 1.24]**	0.13 [−0.56, 0.83]
	Control	−**0.11 [**−**0.65, 0.42]**	0.37 [−0.17, 0.90]

**Repeated Measures ANOVA**			

Main Effect	Occasion	***F* (1, 51) = 22.197, *p* = 0.000, ŋp^2^ = 0.303**	*F* (1, 51) = 2.197, *p* = 0.144, ŋp^2^ = 0.041
	Group	***F* (2, 51) = 4.663, *p* = 0.014, ŋp^2^ = 0.165**	*F* (2, 51) = 1.301 *p* = 0.281, ŋp^2^ = 0.049
Group × Occasion		***F* (2, 51) = 9.270, *p* = 0.000, ŋp^2^ = 0.267**	*F* (2, 51) = 0.027, *p* = 0.973, ŋp^2^ = 0.001
***Post hoc* power**		1.000	0.066

*SPM, Raven’s standard progressive matrices. Cohen’s d-values above 0.5 (medium effect size) and p-values less than 0.05 (significant) are bold values.*

### Qualitative Analysis

#### Data Analyses

We conducted a content analysis of all experimental group feedback with reference to Erlingsson and Brysiewicz (2017), with the primary coder (first author) first coding each feedback independently, and then holding a series of meetings with the second and fourth authors to update the coding approach before finalizing the coding instrument. To establish inter-rater agreement and reliability, the third author was trained in the coding process and independently coded all 27 feedbacks according to the final codebook. Since the focus of this content analysis was to identify the possible impact of MIBMT on users’ lives, three main issues were identified in the process of coding: first, the status of users’ MIBMT use and the problems they encountered; second, what changes occurred in users’ capabilities and their daily lives or academic performance (even if these changes were not definitely attributable to MIBMT use), and whether these changes were supported by evidence; and third, the attitudes of users and their parents toward these changes. The final codebook is shown in Table 15 with corresponding examples for each code and Cohen’s kappa between the two coders (average kappa = 0.87, indicating high reliability; Landis and Koch, 1977).

**TABLE 15:** Codebook of the content analysis in Experiment 2.

Categories	Codes	Reliability (kappa)	Examples
Use of MIBMT	Ongoing use	0.77	Also, not only articles, but she will now actively use this method to remember English words and math formulas.
	Not much used	0.78	It has been about three months since the end of the training, and my child does not usually use the mnemonics learned in the training.
	Forget to use	1.00	However, he also said that what he did not do well was that he sometimes forgot to use this method to memorize.
	Forget the method	1.00	Later I noticed and asked him if he did not use the memory method. He admitted that this method is good but has not used it; he is not unwilling to use the method, but it has slowly fade away.
Problems when using MIBMT	Slow to enter the state	1.00	It takes him a long time to get into the state of mind when memorizing.
	Strong original habit	0.78	I have observed that my child is still too strong in his old habits, and many times I have to remind him before he consciously applies the new memory method.
	Overly busy	1.00	Later, when the midterm exams were approaching, there was a lot of homework, and he was busy with various school assignments every day until 10:30 p.m. He couldn’t finish them, and sometimes he had to stop doing schoolwork to ensure a certain amount of sleep. During that time, both we (parents) and him (child) were busy, and the mnemonic method could not be used at all.
Achievements	General situation	0.78	Overall, she is getting better in all aspects now.
	Chinese	0.78	My child also uses this method to recite ancient poems every day, and the ones that are well understood by his age group can be memorized after reading them only once, while the long poems and those that are not well understood have to be read two or three times. In three months, he has learned a lot of poems beyond the classroom, and the results are remarkable.
	English	1.00	English words are now being memorized very quickly. He used to learn phonetic symbols very well, and now with good methods, it is even better. He even got a perfect score in the recent midterm exam.
	Mathematics	0.91	Math has also improved. He used to get only about 60 or 70 points on the test, sometimes he used to get 50 or 60 points, but now it is much higher than before, and there is no need to retake the test.
	Quality of homework	0.72	He also finishes his homework faster now, and the accuracy rate is much higher than before.
	Speed of learning to play musical instruments	1.00	He told me that during the weekly ukulele lessons, he was able to quickly form a picture of the music scores in his head using this method, and memorized them immediately.
	Others	0.84	For example, last week they had to learn new radio gymnastics, and he said he used this mnemonic method to imprint the pictures of the movements one by one in his mind, and memorized them quickly.
Capabilities	Memorization	0.76	My child has made great progress recently and has been using this method. He can now memorize a short English text after reading it only once or twice.
	Concentration	0.70	The most obvious change I can see in him now is that his concentration has improved.
	Carefulness	1.00	My child used to be more careless, but now he has become better.
	Self-discipline	0.74	After the training, my child’s self-discipline has improved.
	Expression	0.78	I think that with long-term exercise and application, the expression skills will become increasingly better.
	Reading skills	0.89	My child is now able to read using pocket time. His reading has become increasingly faster and the reading content is slowly changing from comic books to literature works!
	Writing skills	1.00	In fact, when I think about it, my child also has made a lot of progress in writing. He now has a framework in his mind before writing, is more organized, and basically gets A’s.
Participants’ feelings	Increase in motivation	0.81	I still remember when my child went to piano lessons after the training. He came back and told me that he loved to play the piano; he used to be very resistant to it.
	Happy	0.76	For example, she would come home and happily tell me that she had applied her newly learned memory method at school and recited everything correctly.
	Self-confidence	1.00	He is becoming more confident in his learning and knows what he wants and what he should do.
	Low motivation	1.00	His motivation to learn is still insufficient and he cannot sink his teeth into his work.
	Rebellious	0.78	My child is now a bit rebellious and sometimes like to confront adults. I do not know why he is being like this. He understands what is good but does not apply it.
	Stressful	1.00	I also communicated with my child, who said that when he saw my face while reciting, he was afraid to go on.
Parents’ feelings	Grateful	0.91	My child and I have benefited a lot from this training, and I would like to thank the teacher for bringing in a new method of memorization!
	Surprised	0.75	He usually scores between 70 and 90 on the quizzes, but scored 94 on the test after the training, which was the biggest surprise for me.
	Satisfied	0.70	Overall, I think the child is in a great place.
	Increased expectations	0.87	After this training, both the child and we have gained a lot, and we hope that he can use this mnemonic method to learn better in the future.
	Openness in parenting	1.00	I used to be very strict with my child, but now I generally let him manage his own studies.
	Parent-child harmony	0.78	Although she used to study well, now that she has an effective method, she feels less overwhelmed by studying and does her homework more easily. With higher efficiency, she has more time to do things she is interested in and has a better understanding of us as parents, and does not feel that we are always making her study.
	Confused	1.00	I am puzzled as to why my child is not using it. Is it because he did not master the mnemonic during the training or is there something wrong?
	Distressed	0.78	Sometimes as a parent it is really tiring and distressing.
	Looking forward to improvement	1.00	I want my child to continue practicing so that this method comes naturally to him and he could have a big improvement in his studying.
	Evidences	0.78	⋅Specific examples: Usually I let my child use this method to memorize, and now he can memorize more than 70 words after reading them once, and has memorized 150 ancient poems in the past few months. ⋅Child’s self-report: Not to mention the memorization, he told me that the images in his brain are becoming increasingly clear, even the Analects of Confucius that his teacher required to pre-study, he could memorize it with just one reading. ⋅Teacher’s feedback: A few days ago, his piano teacher even praised him for his progress in the past few months and his quick memory. My child said that he was using the newly learned mnemonic method.

#### Results

This section presents the analysis results of the feedback submitted by the parents of experimental group members. Of the total feedback received (27), 13 parents mentioned children’s use of MIBMT. In terms of composition, 20 feedback forms contained only positive content, 6 contained both positive and negative content, and 1 included only negative content. Table 16 shows all positive feedback regarding the improvement in users’ competencies, while Table 17 shows all positive feedback regarding academic achievements. The negative feedback is presented in Table 18. It shows the number of feedback forms that reported a change in the corresponding items among all the feedback received from the parents of the experimental group participants (*n* = 27). The numbers in parentheses represent the number of feedback forms received from parents of the high-use group among all the feedback that reported a change in a particular item.

**TABLE 16:** Theme 1: positive feedbacks after training in Experiment 2: improvement of capabilities (the numbers in parentheses represent high-use groups).

Capabilities	Total	Use of MIBMT (Ongoing use)	Evidences
Memorization	23 (11)	12 (5)	20 (9)
Concentration	8 (2)	1	7 (2)
Carefulness	2 (1)		1
Self-discipline	8 (3)	2	4 (2)
Expression	2	1	2
Reading skills	6 (3)	1	3 (2)
Writing skills	2 (2)	1	3 (2)

*N = 27 (High-use group: n = 11).*

*MIBMT, mental-imagery-based mnemonic training.*

**TABLE 17:** Theme 1: positive feedbacks after training in Experiment 2: achievements (the numbers in parentheses represent high-use groups).

		General situation	Chinese	English	Mathematics	Quality of homework	Speed of learning to play musical instruments	Others
Total		15 (5)	14 (7)	16 (8)	8 (6)	7 (4)	6 (2)	3
Use of MIBMT (Ongoing use)		4 (2)	5 (1)	7 (3)	2	1	2 (1)	2
Evidences		8 (4)	11 (5)	12 (6)	6 (4)	4 (2)	5 (2)	2
Capabilities	Memorization	3 (2)	12 (6)	14 (7)	2 (1)	1 (1)	4 (2)	3
	Concentration	2 (1)				2		
	Carefulness				1 (1)			
	Self-discipline	2 (1)	1	1 (1)		3 (1)		
Participants’ feelings	Increase in motivation	1 (1)	1 (1)	2 (1)			2 (2)	
	Happy		1	2			1	1
	Self-confidence	1 (1)		2 (1)				
Parents’ feelings	Grateful	2 (1)	1 (1)	1 (1)			1 (1)	
	Surprised	1	1		1 (1)			
	Satisfied	3 (2)	1 (1)	1 (1)	2 (1)	1 (1)		
	Increased expectations	3 (1)						
	Openness in parenting	1 (1)				1 (1)		
	Parent-child harmony	1 (1)	1 (1)	1 (1)				

*N = 27 (High-use group: n = 11).*

*MIBMT, mental-imagery-based mnemonic training.*

**TABLE 18:** Theme 2: negative feedbacks after training in Experiment 2.

Use of MIBMT		Ongoing use	Not much used	Forget to use	Forget the method
	Total	1	3	2	1
Problems when using MIBMT	Slow to enter the state		1		
	Strong original habit		1	1	
	Overly busy				1
Participants’ feelings	Low motivation		1		
	Rebellious		2		
	Stressful	1			
Parents’ feelings	Confused		2		
	Distressed		2		
	Looking forward to improvement			1	1

*N = 27.*

*MIBMT, mental-imagery-based mnemonic training.*

As shown in Table 16, 23 of the 27 parents reported an increase in their children’s (i.e., participants in the experimental group) memorization skills, and 12 of them stated that their children used MIBMT in general; for example, one feedback stated, “Also, not only articles, but she now actively uses this method to remember English words and math formulas.” Another 20 reports gave evidence (specific examples, feedback from schoolteachers, etc.) when describing memory growth. Improvement in concentration and self-discipline was the second most frequently provided feedback, followed by improvement in reading skills. A small number of parents also reported growth in carefulness, expression, and writing skills. The numbers in parentheses in Table 16 represent the number of children in the high-use group, and it can be seen that for all the children who indicated that they used MIBMT more, their parents reported an improvement in their memorization skills (100%), compared to the low-use group (75%). This is consistent with the results of the quantitative analysis and supports the possibility that MIBMT has a positive effect on memory and that more frequent use leads to better results.

Regarding the improvement in academic achievements, referring to Table 17, it can be seen that positive changes were mainly observed in general, and in Chinese and English. Improvements in mathematics, the quality of homework, and the speed of musical instrument learning were also reported. Concerning English, the most frequently reported language, about half of the parents mentioned that their children used MIBMT to memorize English-related knowledge; for example, one report stated, “She also used this method to memorize English and her English test scores improved. She used to do exceptionally poorly on her exams, but she scored 90 on the recent exam, and she was thrilled.” Almost all of those who reported improvements in English and Chinese mentioned an increase in their children’s memory skills in these subjects. In addition, these positive improvements were accompanied by a positive change in mood for both children and parents. Parents mentioned an increase in children’s motivation and reported that they were more happy and confident. As for parents’ mood, some reports mentioned gratitude for the training, as well as surprise, satisfaction, expectation, openness to parenting, and improved parent–child relationships. These data suggest that MIBMT may play a greater role in subjects that require memorization, such as Chinese and English, but it also has the potential to be applied in mathematics or instrumental learning. These positive changes in academic achievements can have a positive impact on both parents as well as children.

Regarding the feedback that reported negative content, referring to Table 18, a total of three reports mentioned that children did not use MIBMT much and attributed it to the slow uptake of MIBMT and strong original habit. Also, parents observed that their children showed low motivation and resistance, which led to parents’ feelings of confusion and distress. There were two reports of children forgetting to use MIBMT due to strong original habit and one report of a child forgetting how to use MIBMT after a period of time due to being too busy. However, the parents were positive and expected their children to improve in the future. The only feedback that reported using MIBMT but with poor results mentioned that the child felt stressed when reciting in front of the parents but would do better when reciting alone. In general, the observed problems with MIBMT use are more likely to be attributable to the children’s emotional state (resistance, nervousness, etc.) and the effects of the original memorization habits.

### Discussion

The purpose of Experiment 2 was threefold: to re-examine the extent of MIBMT transfer effects, re-examine the sustainability of its transfer effects, and further examine the factors that may affect the effectiveness of training.

In near transfer (Stroop task and non-word recall task), there was an improvement in the performance of all participants in the Stroop task; in the non-word recall task, there was an improvement in the performance of only the experimental group. The frequency of daily use showed that participants who could use the MIBMT more actively in their daily lives showed greater improvement on the non-word recall tasks related to LTM, with a long-term maintenance effect. Since the Stroop task is a measurement task that focuses only on the cognitive control capacity of WM (Pugin et al., 2014), we found no evidence that MIBMT enhances cognitive control capacity. By contrast, the results indicated that the MIBMT improved and maintained users’ performance on tasks associated with LTM capacity.

As for far transfer (arithmetic skills, mental rotation, and SPM tasks), no significant effects were observed from MIBMT. In the Gf-related SPM task, although a significant group × occasion interaction was observed in RT, this may be the result of different baselines between groups. The current data are not sufficient to demonstrate that MIBMT improves user performance in SPM tasks.

In the content analysis, most of the feedback reported positive changes in users’ academic achievements. In the near transfer, increases in users’ memorization and improvements in performance in subjects closely related to memory (Chinese, English) were most frequently reported by parents of participants in the high-use group. As a possible far transfer, feedback also mentioned achievement gains in areas such as mathematics, homework quality, and musical instrument learning, as well as improvements in abilities such as concentration, self-discipline, and reading skills. Although there is no direct evidence that these changes are necessarily causally related to the use of MIBMT, this information still provides a reference for further understanding of the effects of MIBMT.

## General Discussion

The results of the quantitative analysis of this study are listed in Table 19. MIBMT is a cognitive training method that focuses on improving three limitations of current mainstream cognitive training: difficulty in far transfer, the need for equipment such as computers and musical instruments, and the difficulty in continuous use of the training in daily life. MIBMT consists of mindfulness and imagery training, does not require equipment, and is easy to use continuously. The main purpose of this study was to investigate the potential of MIBMT, specifically, the transfer range of the MIBMT effect, maintainability, and the factors that affect it. We first assess the immediate and maintenance effects of MIBMT in near transfer, then evaluate the immediate and maintenance effects of MIBMT in far transfer, analyze the influencing factor, and finally discuss the limitations and potential developments of this study.

**TABLE 19:** Summary of experiment results.

		After training	Maintenance
Experiment 1 (1 year)	**Near-transfer task**		
	Digit span	N	N
	Non-word recall	M-High>M-Low>Control	M-High>M-Low>Control
	**Far-transfer task**		
	Arithmetic skills	N	N
	Mental rotation	N	N
	**Near-transfer task**		
Experiment 2 (3 months)	Stroop	N	N
	Non-word recall	M-High>M-Low>Control	M-High>M-Low>Control
	**Far-transfer task**		
	Arithmetic skills	N	N
	Mental rotation	N	N
	SPM	N	N

*N, none of the effects of MIBMT/potential impact factors were observed; MIBMT, mental-imagery-based mnemonic training; SPM, Raven’s standard progressive matrices; M-High, MIBMT-high-use group; M-Low, MIBMT-low-use group.*

### Discussion of the Near-Transfer Effect

This study examined the role of MIBMT in improving the WM and LTM performance of users through Experiments 1 and 2. Experiment 1 used a digit span task that focused only on measuring the storage capacity of WM, while Experiment 2 used a Stroop task that focused on measuring the cognitive control capacity of WM. Our findings showed that the use of MIBMT did not improve participants’ performance in either Experiment 1 or 2. As mentioned earlier, there are various models of WM (e.g., Baddeley, 2003; Unsworth and Engle, 2007), but the two features they have in common are limited storage and cognitive control (Schwaighofer et al., 2015), the two most important aspects of WM. In this study, the effects of MIBMT on WM storage capacity were tested in Experiment 1, while Experiment 2 tested attentional control; however, no significant results were found. This suggests that the effect of MIBMT on the WM capacity of users is limited, and if there is any effect on users’ other abilities, the cause of the effect is also likely to be unrelated to WM.

Both Experiments 1 and 2 showed that the use of MIBMT had a significant effect on the improvement of participants’ LTM; according to the results of Experiment 1, this effect was sustained for one year. Further, the effect was greater for individuals who used it more frequently. The results of the quantitative analysis were further supported by the content analysis in Experiment 2, which showed that such improvement in memorization is likely to have a positive effect on users’ performance in memory-related activities (e.g., Chinese, English, and other subjects requiring memorization). From a mechanistic point of view, although WM as well as increased levels of stimulus encoding and information retrieval can improve LTM performance (Carretti et al., 2007), given that there was no direct relationship between MIBMT use and WM in this study, it is possible that the two steps of MIBMT—converting stimuli into mental images and recollection of the target stimulus in reverse order—increased the level of encoding and information retrieval, which was responsible for the improvement of LTM capacity. Further, according to Unsworth and Engle’s (2007) WM model, WM is supported by two separable cognitive processes: cue-dependent controlled retrieval and task-related recall from LTM resources. Therefore, when LTM is improved, the ability to recall task-related information in WM may also be improved. The results of Experiments 1 and 2 illustrated that MIBMT had a positive effect on LTM in this study; however, there was no significant relationship with WM. Thus, future studies must examine the relationship between MIBMT and task-related recall ability in WM.

### Discussion of the Far-Transfer Effect

We examined the effects of MIBMT on users’ arithmetic ability (arithmetic skills task), spatial ability (mental rotation task), and Gf (SPM task) in Experiments 1 and 2. In both Experiments 1 and 2, the use of MIBMT did not improve participants’ performance on the arithmetic skills task and the mental rotation task. Experiment 2 showed that there were differences in the baseline between the experimental and control groups in the SPM task, which led to the present data not being able to deduce a positive effect of MIBMT on this task either.

However, a part of the feedback in Experiment 2 mentioned improvement in users’ mathematics test scores and reading and writing skills. Therefore, to further clarify the effect of MIBMT on mathematics ability, subsequent studies should take measures such as adding improved task content or increasing the sample size. According to Jola and Mast (2005), it is possible that cognitive processes differ for distinct tasks such as the spatial perception (Linn and Petersen, 1985) and mental rotation tasks. Therefore, it may be insufficient to assess users’ spatial ability on the mental rotation task, and subsequent studies should use different types of spatial ability tasks to further clarify the relationship between MIBMT and spatial ability. Regarding the SPM task, since Gf includes a variety of cognitive abilities such as memory, reasoning, and reading comprehension (Jaeggi et al., 2008), subsequent research should further examine the effects of MIBMT on the improvement or maintenance of cognitive abilities such as reading comprehension, in addition to reasoning ability, by establishing a longer experimental period, adding related tasks.

## Limitations

This study has five limitations. First, several studies related to cognitive training have reported the placebo effect on experimental outcomes (e.g., Simons et al., 2016). In the present study, at the beginning of Experiments 1 and 2, the experimental goal expressed to the control group was to track the development and change of various abilities such as memory in children and adolescents over a 1-year or a 3-month period. This may have encouraged them to raise their subsequent test scores. However, overall, the placebo effect was not completely ruled out, given that the control and experimental groups were not exactly equivalent in terms of motivation level. Nevertheless, according to the theory of selectivity or systematicity of transfer (Chein and Morrison, 2010), task scores should generally improve if the experimental effect comes from the placebo effect alone, which was not observed in this study. Furthermore, the use of a passive control group does not help us to clarify the role each component of MIBMT plays in the overall training effect. Given the complexity of the training program, it is unknown whether mindfulness or MI contributes the most to the effectiveness of MIBMT. In future studies, an active control group using only the mindfulness or MI strategy will help us to verify the specific effects of each component. Moreover, since our participants were adolescents, they would likely experience a fairly steep learning curve; therefore, an active control group would be a better choice for future studies to clarify the effects of MIBMT.

Second, the participants in the experimental and control groups were not randomly assigned. Due to the training time required for MIBMT, we assigned participants who could spare eight days to the experimental group. Although in Experiment 2, all participants were from the same grade level in the same elementary school, and their status and income were essentially at the same level according to the pre-survey, there may still be other confounding variables that could have influenced the results of the experiment. Random grouping might be a better choice in future studies.

Third, even though we expanded the sample size in Experiment 2, baseline inconsistency still appeared in the SPM task. Further expansion of the sample size might improve this problem.

Fourth, the single PTE questionnaire might have been too simple to assess participants’ frequency of MIBMT use in their daily lives. Since the participants were elementary school students, to ensure that they understood the questions correctly, it was specifically stated when the questionnaire was administered that it evaluated the frequency of using MI to encode and recollection of the target stimulus in reverse order to remember content. Therefore, it is accurate to say that the PTE evaluation is more about the frequency of using MI strategies and recollection in reverse order in MIBMT. We did not specifically ask whether the use of these strategies was preceded by mindfulness practice to put oneself in a relatively focused state. This should be clarified in a later study.

Finally, the current research did not clarify the specific mechanisms underlying the benefits of MIBMT. There are various possible explanations for the cognitive processes that might be influenced, such as improved processing speed, improved encoding efficiency, better acquired encoding, chunking or maintenance skills, changes in attentional defenses, and anti-interference capabilities (Morrison and Chein, 2011). Clarifying the effects of MIBMT on each specific part of the cognitive process in future studies will facilitate the assessment of the exact effects of this training modality and the amount of training necessary for MIBMT to yield effects.

## Conclusion

In this study, the effectiveness of MIBMT on LTM and the sustainability of its effect were examined. Frequency of daily use influenced the effects of MIBMT. The results of the content analysis also showed positive effects of MIBMT on LTM and the subjects associated with it. Although the results of the content analysis also showed that MIBMT has the potential to produce far transfer effects in mathematical ability, concentration, self-discipline, and reading skills, these effects and the factors that influence them need further quantitative research.

Unlike other types of cognitive training, MIBMT can be easily applied to daily life post-training. Other forms of training also exploit the features of MI that can promote memory performance, such as digit-image mnemonics (Yin et al., 2015) and the method of loci (Dresler et al., 2017). Pressley (1976) indicated that MI could improve eight-year-olds’ performance in recalling prose. This mnemonic strategy is not universally used as a tool to improve cognitive abilities, as its material specificity limits the occurrence of transfer (Morrison and Chein, 2011). By contrast, MIBMT does not restrict the type of stimulus; it only requires the user to convert the target stimulus into MI and manipulate it while maintaining the image. This may help overcome the limitations of traditional mnemonic strategies in relation to stimulus material specificity, thus improving the effectiveness and generalizability of this kind of training program. As shown in our content analysis, many parents of participants reported that their children used MIBMT effectively to improve their academic performance in subjects that required memorization, such as Chinese, English, and even the memorization of music scores or movements in radio gymnastics. MIBMT was effective in preventing users from making memory errors caused by top–down processing and in helping them acquire more accurate knowledge. In turn, this improved performance can increase the user’s motivation and confidence in their studies and have positive effects such as promoting parent–child harmony.

Overall, our results suggest that MIBMT is an effective cognitive program in enhancing LTM performance that is well adapted to its users’ needs and has the potential for making far transfer. We hope to further clarify the mechanism of MIBMT in future studies, and explore the possibility of applying MIBMT to various fields such as second-language learning and motor skills.

## Data Availability Statement

The data that support the findings of this study are openly available in Open Science Framework at http://doi.org/10.17605/OSF.IO/75JZP, reference number osf.io/75jzp.

## Ethics Statement

The studies involving human participants were reviewed and approved by Ethics Review Committee on Research with Human Participants of Waseda University. Written informed consent to participate in this study was provided by the participants’ legal guardian/next of kin.

## Author Contributions

XL: conceptualization, data curation, formal analysis, methodology, project administration, writing – original draft, software, and visualization. ES: conceptualization, funding acquisition, supervision, validation, and writing (review and editing). QC: conceptualization, investigation, and resources. YK: formal analysis and writing (review and editing). All authors contributed to the article and approved the submitted version.

## Conflict of Interest

The authors declare that the research was conducted in the absence of any commercial or financial relationships that could be construed as a potential conflict of interest.

## Publisher’s Note

All claims expressed in this article are solely those of the authors and do not necessarily represent those of their affiliated organizations, or those of the publisher, the editors and the reviewers. Any product that may be evaluated in this article, or claim that may be made by its manufacturer, is not guaranteed or endorsed by the publisher.

## Footnotes

1 The differences in developmental and gender effects across all tasks were non-significant. Therefore, we described the results of the analysis for all participants, regardless of their age or gender.
